# A Novel Protein Kinase-Like Domain in a Selenoprotein, Widespread in the Tree of Life

**DOI:** 10.1371/journal.pone.0032138

**Published:** 2012-02-16

**Authors:** Małgorzata Dudkiewicz, Teresa Szczepińska, Marcin Grynberg, Krzysztof Pawłowski

**Affiliations:** 1 Nencki Institute of Experimental Biology, Polish Academy of Sciences, Warsaw, Poland; 2 Institute of Biochemistry and Biophysics, Polish Academy of Sciences, Warsaw, Poland; 3 Warsaw University of Life Sciences, Warsaw, Poland; American University in Cairo, Egypt

## Abstract

Selenoproteins serve important functions in many organisms, usually providing essential oxidoreductase enzymatic activity, often for defense against toxic xenobiotic substances. Most eukaryotic genomes possess a small number of these proteins, usually not more than 20. Selenoproteins belong to various structural classes, often related to oxidoreductase function, yet a few of them are completely uncharacterised.

Here, the structural and functional prediction for the uncharacterised selenoprotein O (SELO) is presented. Using bioinformatics tools, we predict that SELO protein adopts a three-dimensional fold similar to protein kinases. Furthermore, we argue that despite the lack of conservation of the “classic” catalytic aspartate residue of the archetypical His-Arg-Asp motif, SELO kinases might have retained catalytic phosphotransferase activity, albeit with an atypical active site. Lastly, the role of the selenocysteine residue is considered and the possibility of an oxidoreductase-regulated kinase function for SELO is discussed.

The novel kinase prediction is discussed in the context of functional data on SELO orthologues in model organisms, FMP40 a.k.a.YPL222W (yeast), and ydiU (bacteria). Expression data from bacteria and yeast suggest a role in oxidative stress response. Analysis of genomic neighbourhoods of SELO homologues in the three domains of life points toward a role in regulation of ABC transport, in oxidative stress response, or in basic metabolism regulation. Among bacteria possessing SELO homologues, there is a significant over-representation of aquatic organisms, also of aerobic ones. The selenocysteine residue in SELO proteins occurs only in few members of this protein family, including proteins from Metazoa, and few small eukaryotes (*Ostreococcus*, stramenopiles). It is also demonstrated that enterobacterial mchC proteins involved in maturation of bactericidal antibiotics, microcins, form a distant subfamily of the SELO proteins.

The new protein structural domain, with a putative kinase function assigned, expands the known kinome and deserves experimental determination of its biological role within the cell-signaling network.

## Introduction

Selenoproteins are an intriguing evolutionary creation, characterised by the presence of an atypical aminoacid residue, selenocysteine. Maintaining the machinery for selenocysteine synthesis and incorporation just for the sake of a handful of proteins is costly [1], also the requirement for selenium acquisition from environment poses difficulty. However, advantages of selenocysteine (Sec, U) as compared to cysteine (Cys, C) may partly offset these problems. Among the unique properties of selenocysteine, instead of cysteine in enzymatic active sites, higher nucleophilicity of Sec versus Cys, higher oxidoreductase efficiency, and lower p*K*_a_, have been cited [1].

Human selenoproteins are encoded by 25 genes, and most of those with known functions are oxidoreductases with a selenocysteine being in the active site. A few human selenogenes are functionally uncharacterised [2]. In addition, we suppose that in the uncharacterised selenoproteins, a selenocysteine residue conserved in evolution is not very likely to be just a troublesome decoration. A catalytic or regulatory function for this residue, and the protein as a whole, seems more reasonable. Hence, we undertook a structural and functional prediction study for the human selenoprotein O (SELO), one of the very few uncharacterised selenoproteins in humans. The human SELO (NCBI gi: 172045770) has been predicted to be a selenoprotein by a bioinformatics approach and confirmed to be an expressed selenoprotein by Gladyshev and co-workers [2]. As outlined in the Results section, not all SELO family proteins identified by us are selenoproteins, yet we use the SELO name for the entire family for consistency. SELO selenoproteins have a single selenocysteine residue while those family members that are not selenoproteins, usually have a cysteine residue instead in the corresponding position. In most eukaryotes and many bacteria, SELO is present as a single-copy protein, while duplicate copies in many metazoans and a few bacteria exist.

Incidentally, a few years ago Koonin and colleagues proposed SELO among the top ten most-wanted ”unknown unknowns”, when discussing the proteins of unknown structure and function posing exciting conceptual challenges for structure predictors, based on phyletic spread [3]. The same authors reiterated that list just recently, indicating “no news” for SELO [4].

Protein kinase-like (PKL) proteins are a huge clan of regulatory/signalling and biosynthetic enzymes [5], [6]. They regulate most processes in a living cell, phosphorylating a wide spectrum of substrates: proteins, lipids, carbohydrates, and smaller molecules. Besides PKL kinases, other kinase families are known, functionally related, but of dissimilar structures [7], [8]. Most PKL proteins feature a well-conserved structural scaffold, and a conserved active site. These “classic” protein kinases number more than 500 in the human genome, and due to their regulatory functions, they are among the most popular drug targets [9], [10], [11]. The importance of kinases in biology is reflected by several kinase-dedicated databases, e.g. Protein kinase resource [12], Kinomer [13], Kinbase [14], Kinase Knowledgebase [15], cataloguing structural, chemical and functional information.

A large group of PKL proteins, lacking elements of the archetypical catalytic site, have been termed pseudokinases and believed to be inactive. However, these proteins are recently “being rethought” [16], [17]. Examples appear of pseudokinases that retain some residual phosphorylation catalytic capability despite an “incapacitated” active site, like Erbb3, a disabled kinase with residual activity lacking the “essential” HRD motif. In Erbb3, Asp is replaced by Asn, and alternative catalytic mechanism is proposed [18]. Zeqiraj and co-workers describe four types of pseudokinases: Predicted pseudokinases, Pseudokinases, Low activity kinases, Active pseudokinases [17]. Here, we demonstrate the evidence that SELO definitely belongs to the first type (predicted pseudokinase), and further present arguments that SELO is likely to be type three or four (Low activity kinase or Active pseudokinase).

Protein domains of unknown function (DUFs) constitute a substantial part of the proteome of any organism [19]. Structure determination efforts for DUFs inform us that “DUF families likely represent very divergent branches of already known and well-characterized families“[20]. Thus, remote homology detection/structure prediction methods are now a routine approach for proteome annotation [21]. However, in some cases, supervised approach by expert users still offers added value to automated annotation pipelines. Precedents include detection of a metalloprotease domain in proteins claimed earlier to be ion channels [22], identification of a peroxiredoxin-like domain in well-studied tumor-implicated proteins [23], and functional annotation of eight DUFs [24].

In this paper, we first present the kinase-like structural prediction for the SELO family. Then, we discuss the relevance of the structural predictions for the predicted molecular function. Furthermore, we analyse the phylogenetic spread of the *SELO* genes, and the characteristics of the bacterial and archaeal species possessing the SELO domain proteins. We also summarise and analyse the available functional data for SELO as well as its most studied orthologues, namely those from yeast and *Escherichia coli*. In the end, we present evidence for kinship between the SELO family and mchC proteins present in a few enterobacterial strains and involved in maturation of the bactericidal antibiotics, microcins.

## Results

### Identification and structure prediction of the SELO domain

In order to predict structure for the SELO protein, the FFAS03 [25] method was used, yielding consistent, albeit of borderline significance, predictions of kinase-like superfamily in the SCOP database (http://scop.mrc-lmb.cam.ac.uk/scop/) and various families of the kinase-like clan (CL0126) in the Pfam database (see Table 1 and Table S1). Also, HHpred [26] ascribes the SELO family to kinase-like proteins (see Table 1 and Table S1). More specifically, the FFAS03 and HHpred structure prediction methods consistently flagged the Kdo Lipopolysaccharide kinase (Kdo/WaaP) family proteins (PF06293) [27], APH Phosphotransferase enzyme family (PF01636) [28] and various representatives of the “classic” protein kinase family (PF00069). In the SCOP database, the fold d.144, Protein kinase-like (PK-like, PKL) dominated among the hits as the closest structural match for the SELO domain. Similar results were obtained for the *Escherichia coli* and yeast orthologues.

**Table 1:** Structure predictions for SELO proteins.

SELO_HUMAN, FFAS predictions				
Z Score	HIT ID		Hit name	Organism
**Pfam**				
−7.220	PF06293.7		Lipopolysaccharide kinase (Kdo/WaaP) family (PKL clan)	-
−5.920	PF00657.15		GDSL-like Lipase/Acylhydrolase (SGNH_hydrolase clan)	-
−5.790	PF07579.4		Domain of Unknown Function (DUF1548)	-
−5.330	PF01163.15		RIO1 family (PKinase clan)	-
**SCOP**				
−7.050	1zar	d.144.1.9	Rio2 serine protein kinase C-terminal domain	*Archaeoglobus fulgidus*
−5.900	1nd4	d.144.1.6	Aminoglycoside 3′-phosphotransferase IIa (Kanamycin kinase)	*Klebsiella pneumoniae*
**PDB**				
−6.130	1e7u		Phosphatidylinositol 3-kinase catalytic subunit	*Sus scrofa*

SELO_HUMAN, HHpred predictions				
Pfam				
Pval	HIT ID		Hit name	Organism
0.000008	pfam06293		Kdo (PKinase clan)	-
0.00041	pfam01636		APH phosphotransferase (PKinase clan)	-
0.00017	pfam07579		DUF1548	-
**SCOP**				
0.0024	1zyl	d.144.1.6	RdoA kinase	*Escherichia coli*

Assignments of human SELO protein (gi: 172045770), to PDB, SCOP and Pfam using FFAS and HHpred methods. Top hits shown for each combination of method, query protein, and database. For Scop hits, d.144 denotes members of Protein kinase-like (PK-like) fold. Prediction for other SELO proteins are shown in Table S1.

The kinase-like fold predictions were obtained for the central region of human SELO protein, with alignments to the known kinase-like structures spanning the stretch from approx residue 120 to 470. For the remaining SELO regions (residues 1–119 and 471–669), no structural predictions were obtained nor homologous sequences outside the SELO family were found. The SELO alignments to different kinase domain hits did not always cover the whole domain, yet together they did cover most of the domains. For example, the FFAS alignment for SELO and PKA kinase included the 47–283 region of PKA (67% of the kinase domain sequence). Likewise, HHpred alignment for SELO and the *E. coli* RdoA kinase covered 93% of the RdoA kinase domain as defined in the SCOP database.

Structural predictions can be validated by predictions for distant homologues using different methods. Indeed, using the sequence of a SELO homologue, the “conserved hypothetical protein [*Marinomonas* sp. MED121]”, gi:86163056, one obtains in five PSI-BLAST iterations significant similarity to a Ser/Thr protein kinase of the PIM subfamily [*Saccoglossus kowalevskii*] (XP_002739315.1), E-value 2E-3, with sequence identity 13%, in a 296 residue-long alignment. The Saccoglossus protein is a close homologue of human proto-oncogene Ser/Thr-protein kinase PIM1.

It has to be borne in mind that structure predictions are not automatically extendable to functional predictions. Thus, functional meaning of the structure assignment for SELO proteins will be discussed further down.

### Survey of SELO proteins, phylogenetic spread, the relationship of the SELO family to the rest of the protein kinase-like clan

Standard BLAST searches for distant SELO protein homologues did not bring up any sequences other than those recognisable as SELO. The SELO protein family is recognised in the Pfam database as one of the “Domains of unknown function”, UPF0061 (PF02696), annotated as “Uncharacterized ACR, ydiU/UPF0061 family”. In the Clusters of Orthologous Groups (COG) database [29], bacterial SELO proteins are combined in the group COG0397.

In order to survey SELO and similar proteins in the sequence “hyperspace”, we used the conceptually similar Saturated BLAST [30] and HHsenser [26] search tools with the central part of the human SELO protein (NCBI gi: 1699163, region 190–470) as the query, using the standard search parameters (see Methods section for details). These procedures, which perform cascades of PSI-BLAST or HHsearch searches, respectively, using representative significant hits as queries in subsequent iterations, yielded several hundred “hit” sequences from the nr and env_nr databases.

Since such a search can easily “drift out” of the original (SELO) sequence family, the hit sequences were checked for the presence of proteins that could be assigned to already known structural domains using the well-established Pfam domain classification system [31] that distinguishes protein domain families, sometimes grouped in clans. In the total hit sequence population, none were easily assigned (using the standard HMMER tool on the Pfam database) to any sequences but the UPF0061 family.

Out of 859 HHSenser “hit” sequences, removal of redundancy at 70% sequence identity threshold yielded a set of 143 sequences which was treated as a representative set of the SELO domain proteins. Out of the 143 sequences, those with assigned organism of origin belonged to all three branches of the tree of life: Bacteria (43 sequences), Archaea (1 sequence) and Eukaryota (33 sequences). The rest (46 sequences) were assigned to marine metagenome, i.e. environmental samples from Sargasso Sea [32], most likely representing bacteria as well.

The bacterial representative SELO proteins included family members from most bacterial taxons: gamma-Proteobacteria (24 sequences), alpha-Proteobacteria (6 sequences), beta-Proteobacteria (5 sequences), Cyanobacteria (4 sequences), two sequences from Bacteroidetes, epsilon-Proteobacteria, Actinobacteria, and Firmicutes, as well as single sequences from Verrucomicrobia, delta-Proteobacteria, Enterobacteria, and Spirochetes. The archaeal SELO proteins are found in Euryarchaeota. Additional BLAST analyses revealed SELO homologues in several more bacterial phyla (see the SELO phylogeny section below).

An independent query for SELO homologues in the Integrated Microbial Genomes (IMG) system [33] confirmed an uneven distribution of SELO in the three domains of life. A BLAST search starting from ydiU protein, the *E. coli* SELO homologue, and using the cutoff of E-value of 0.005, revealed SELO was present in three archaeal genomes out of 107, 1101 bacterial genomes out of 2780 and 79 eukaryotic genomes out of 121 (3%, 40% and 65%, respectively).

**Figure 1:**
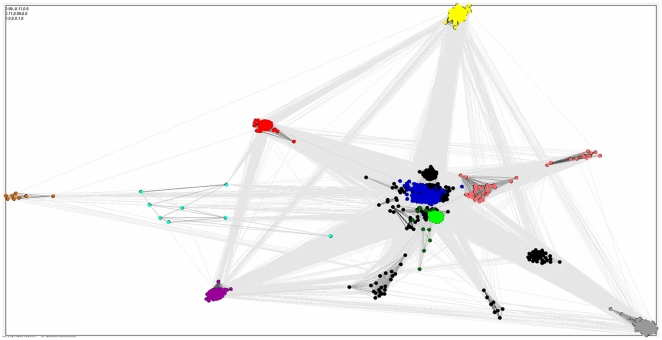
CLANS graph visualizing PSI-BLAST-detected significant (dark grey) and sub-significant (light grey) similarities among protein kinase-like proteins. Red: UPF0061 (SELO) family, Light green: pkinase and pkinase_Tyr, Dark blue: APH phosphotransferase, Magenta: PIP5K, Grey: alpha_kinase. Pink: PI3_PI4, Light blue: Act_Frag_cataly, Yellow: PPDK_N, Dark green: Kdo, Orange: UL97.

The relationship of the SELO family to the PKL clan can be visualized using a graph-based approach, the CLANS algorithm [34]. The CLANS graph visualizes PSI-BLAST-detected significant and sub-significant similarities whereas proteins, represented as dots, are grouped using “attractive forces” dependent on sequence similarities. SELO appears to be a valid member of the clan (see Fig. 1), with strong links to central families (pkinase and pkinase_Tyr, the “classic” threonine/serine and tyrosine protein kinases), but also to most of the other families. In the CLANS analysis, SELO family (UPF0061) is linked both to distant members of the kinase-like clan (e.g. viral UL97 kinases, PF06734 [35]) and to known kinase families that were not assigned as kinase-like clan members previously (e.g. alpha-kinases, Alpha_kinase (PF02816) [36]).

Often, domain fusion events shed light on function of the fused domain. For the SELO kinase-like domain, three proteins with extra domains were identified by HMMER, in *Monosiga brevicollis* MX1 (gi: 167537910), in *Schistosoma mansoni* (gi: 256073786) and in *Laccaria bicolor* S238N-H82 (gi: 170098891). However, these proteins have no corresponding multidomain homologues and are likely the artifacts of genome annotation.

No transmembrane regions were detected in SELO proteins using standard methods. The TargetP and MultiLoc methods predict human and other vertebrate SELO proteins to contain an mTP, mitochondrial targeting peptide, and therefore to be mitochondrial proteins. However, for other eukaryotes, various locations are predicted, including cytoplasm and secretory pathway. For the yeast SELO homologue, the FMP40 protein, mitochondrial localisation has been experimentally determined by several high-throughput studies, and identified as one of a few Tyr-nitrated mitochondrial proteins [37].

### SELO phylogeny, the selenocysteine region in SELO

**Figure 2:**
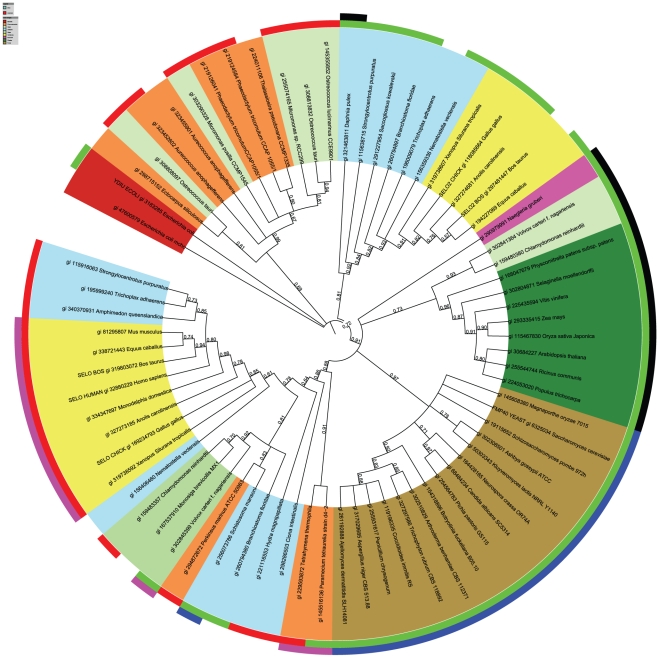
Phylogenetic tree (PhyML) of the representative SELO proteins. Tree branch coloring: Red: Bacteria, Dark green: Plants, Light green: Green algae, Orange: Chromoalveolata, Magenta: Excavates, Yellow: vertebrates, Blue: non-vertebrate Metazoa, Brown: Fungi. Inner ring: presence of Cys or Sec at C-terminus: CXX> motif: green; UXX> motif: red. Outer ring: presence of additional Cys residues prior to the [CU]XX> motif: CXX[CU]XX>: magenta, CX[CU]XX>: blue, CX(3–10)[CU]XX>: black. Identifiers: NCBI gi numbers. The tree is built using the alignment shown in Figure S3.

To explore relationships between eukaryotic SELO proteins, 73 SELO representatives were arbitrarily selected from genomes of diverse organisms spanning all major eukaryotic taxons that possess SELO family members. Also, two sequences from he bacterium *E. coli* were included. The eukaryotic SELO phylogenetic tree includes five main branches (Fig. 2). Firstly, a branch with sequences from very diverse organisms that can be loosely labeled together as marine picoeukaryotes. These include sequences from green algae: *Micromonas* and *Ostreococcus*, stramenopiles: *Aureococcus*, the brown alga *Ectocarpus* and the diatoms *Thalassiosira* and *Phaeodactylum*. Secondly, the tree includes a branch with metazoan sequences, homologues of vertebrate SELO2 (see below for definition) and sequence from other taxons: *Cnidaria* (*Nematostella*), hemichordates (*Saccoglossus*), plocozoans (*Trichoplax*), echinoderms (*Strongylocentrotus*), crustaceans (*Daphnia*). Branches 3, 4, and 5 are grouped together with strong bootstrap support of 0.91. The third branch contains plant and green algal proteins. The fourth one is mostly composed of fungal sequences. The fifth branch not only includes metazoan sequences, homologues of SELO, but also sequences from green algae (*Monosiga, Volvox, Chlamydomonas*), and alveolates (*Perkinsus, Paramecium, Tetrahymena*). Separate in the super-branch (3,4,5) is the sequence from the excavate *Naegleria*. Two bacterial sequences, ydiU and mchC from *E. coli*, remain an outgroup. Notable is the absence of the *SELO* gene in all nematodes including *C. elegans* and in most arthropods, including *Drosophila* and all insects. An exception is the tick *Ixodes* and crustaceans, e.g. *Daphnia*.

The similarities of metazoan SELO domains (see the phylogenetic tree in Fig. 2) suggest that (a) the common ancestor of Metazoa had a duplicated *SELO*-like gene (most Metazoa analysed, including the simple plocozoan *Trichoplax*, have two paralogues of the *SELO* gene) and (b) in some lineages, e.g. humans, the second gene, *SELO2*, was lost. The *SELO2* gene is present throughout Metazoa, including *Gallus* [Entrez Gene: LOC420491], *Canis* [Entrez Gene: LOC607965], *Equus* [Entrez Gene: LOC100065515], and *Bos* [Entrez Gene: LOC100297097]; however, it is missing from primates, rodents, and many other mammals. The *SELO* and *SELO2* genes are not close homologues, e.g. in *Bos* the proteins share as little as 36% sequence identity over 550 residues. Here, we have introduced the unofficial gene symbol *SELO2* in order to denote the homologue of *SELO* present throughout Metazoa, although SELO2 is not a selenoprotein.

*SELO* gene duplication is also observed in green algae, e.g. *Ostreococcus*, *Chlamydomonas* and *Volvox* (see branches 3 and 5 in Fig. 2) as well as stramenopiles, *e.g. Aureococcus, Phaeodactylum* (see branch 1 in Fig. 2); however, these duplications seem to be independent of the *SELO/SELO2* duplication in Metazoa. The duplicated genes are relatively distant, and the sequence identity for the aligned paralogue pairs is 26%, 41%, 36%, 44% and 58% in *Ostreococcus*, *Chlamydomonas*, *Volvox*, *Aureococcus* and *Phaeodactylum*, respectively.

Among the five main eukaryotic phyla as delineated by Koonin [38], *SELO* is absent from *Rhizaria* and present in representatives of the other four phyla: Unikonts, except *Amoebozoa*, Excavates (but only in the amoeboflagellate *Naegleria gruberi*), Plantae and Chromoalveolates, except apicomplexans. Thus, *SELO* is probably a pan-eukaryotic gene that has been lost from a number of genomes.

A phylogenetic tree including representative sequences from all domains of life as well as the marine metagenomic sequences, unassigned to a taxon, shows a similar picture (see Fig. S1), albeit highlighting the difficulties in elucidating the evolutionary relationships between eukaryotic and prokaryotic SELO homologues. Nevertheless, the most plausible evolutionary scenario would involve *SELO* as an ancient gene in the last universal ancestor of bacteria. Eukaryotes would have gained SELO from the ancestor of the mitochondrial endosymbiont, while a few Archaea would have acquired it via horizontal gene transfer. Accordingly, SELO remains a mitochondrial protein in all the studied eukaryotes. *SELO* gene loss in several eukaryotic and bacterial lineages would have produced the taxonomic spread observed today.

Consistent with the evolutionary hypothesis outlined above, among the prokaryotic phyla, SELO proteins are found in approximately half of the major bacterial taxons, as listed by the Genomic Encyclopaedia of Bacteria and Archaea [39] and the Tree of Life Web Project [40]: *Acidobacteria*, *Actinobacteria*, *Bacteroidetes*, *Chrysiogenetes*, *Cyanobacteria*, *Deinococcus-Thermus*, *Firmicutes*, *Gemmatimonadetes*, *Nitrospira*, *Planctomycetes*, *Proteobacteria*, *Spirochetes*, and *Verrucomicrobia*. However, no SELO homologues were found in *Aquificae*, *Chlamydiae*, *Chlorobi*, *Chloroflexi*, *Deferribacteres*, *Dictyoglomi*, *Elusimicrobia*, *Fibrobacteres*, *Fusobacteria*, *Synergistetes*, *Tenericutes*, *Thermodesulfobacteria*, *Thermomicrobium* and *Thermotogae*. Among Archaea, SELO proteins are found exclusively in several Euryarchaeota species. Of note, a few bacterial genomes, e.g. cyanobacteria, *Acaryochloris* and gamma-proteobacterium *Marinomonas* sp. MED121, have duplicated SELO genes, with relatively low sequence identity (28%). A more divergent case of *SELO* gene duplication produced the *mchC* gene in a small group of gamma-proteobacteria (see the separate mchC section below).

**Figure 3:**
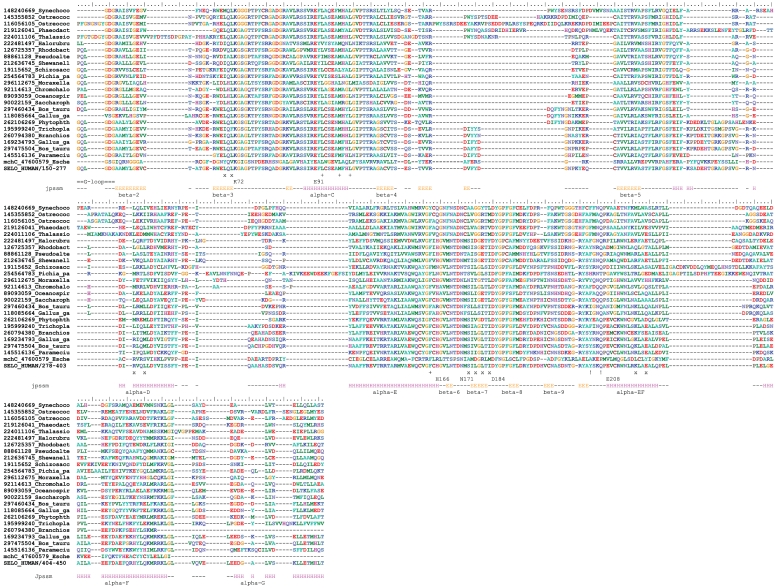
Multiple sequence alignment of selected SELO proteins (representatives of branches identified in the tree in **Fig. 2**). mchC protein is the penultimate sequence. Secondary structure prediction for human SELO protein is shown. Secondary structure elements named as in PKA, according to Knighton [51]. “x” denotes putative residues belonging to the C-spine (catalytic), “+” denotes putative R-spine (regulatory) residues. Exclamation signs denote potential phosphorylation sites in the activation loop. Locations of predicted key catalytic residues shown, in standard PKA numbering (e.g. H166). Identifiers: NCBI gi numbers.

The SELO proteins often possess a Cys-x-x-[Cys/Sec]-x-x-> motif (e.g. CVTUSS> in humans) at the C-terminus, where the “>” character denotes the terminus of the polypeptide. Such motifs are more common than expected by chance (according to Prosite Scan results, http://prosite.expasy.org/, there are 585 observed CxxCxx> occurrences in the SwissProt database versus 281 expected considering average cysteine occurrence; probability of the observed or higher number of CxxCxx> motifs is practically 0). The CxxC motifs, in general, are reminiscent of an oxidoreductase, e.g. thioredoxin, function [41] and are present in majority of thioredoxin-fold proteins [42], [43], [44]. The CxxU motif has been shown in another selenoprotein, the selenoprotein H [45], to correspond with the thioredoxin active site. For selenoprotein H, homologues with CxxC instead of CxxU occur in insects and plants. Similarly, in a number of SELO protein homologues, the CxxUxx> motif is replaced by CxxCxx> or by a single cysteine residue. Out of SELO homologues, cysteine located two positions prior to the C-terminus, Cxx>, is more common than selenocysteine, Uxx> (See Fig. 3 and Fig. S1).

Generally, the location of selenocysteine near the C-terminus of a selenoprotein is a common feature among these proteins, occurring either in CxxU pairs or as single U residues [46]. In the selenoprotein K [47], an Uxx> motif is found, while in selenoprotein S [48], there is an Ux> motif. Both these proteins are involved in stress responses. Selenoprotein P (SEPP) contains an UxUxxx> motif, and TXNRD1, TXNRD2 and TXNRD3 proteins have each U as the penultimate residue (Ux>). In the latter three proteins, thioredoxin reductases [49], also in the lipid hydroperoxidase SEPP [50], selenocysteine is involved in the oxidoreductase function.

A rigorous assessment of the significance of the occurrence of the CxxC/CxxU motif at SELO protein C-termini is not straightforward because of uneven taxonomic sampling of SELO homologues. However, in the representative set of 143 SELO proteins, selected for even coverage of the sequence space (see Methods section), there are 12 proteins with this motif. Although this is a clear minority of SELO proteins, it still is an overrepresentation as compared to a random situation. The binomial test using SwissProt as reference population (585 occurrences in half a million sequences) allows one to estimate the probability of Cxx[CU]xx> motif occurring by chance in the SELO family at less than 10^−18^. Thus, one can postulate that this motif may have a functional role. Also, a generalised [CU]xx> motif occurs in the representative SELO set 111 times, which is a clear overrepresentation (binomial test probability less than 10^−189^).

The CxxC and CxxU motifs in SELO proteins are not evenly distributed on the phylogenetic tree. The Metazoan SELO branch (branch 5 in Fig. 2) features mostly the Cxx[CU]xx> C-terminal motif. The fungal branch (branch 4) is characterised by the CxCxx> motif. The plant branch (branch 3) is characterised by the C-x(3,10)-Cxx> motif. The Metazoan SELO2 branch (branch 2) has usually just the Cxx> motif, while the “marine picoeukaryote” branch 1 has usually the Uxx motif>. The bacterial proteins have usually a Cxx> motif, sometimes augmented by an additional C residue to a CxxCxx> motif or a C-x(3–10)-Cxx> one (see Fig. S1).

### Structure models of the SELO domain, the relevance of the structure predictions for molecular function

Secondary structure predictions and sequence alignments to the known kinase-like structures according to structure prediction results suggest that the SELO domains are composed of an N-terminal smaller lobe (ATP-binding), mostly composed of β-strands, and a predominantly helical, larger, C-terminal lobe (phosphotransfer). In terms of the classic kinase fold nomenclature [51], [52], SELO domain secondary structure order is as following: β-2, β-3, α-C, β-4, β-5, α-D, α-E, β-6, β-7, β-8, β-9, α-EF, α-F, α-G, see Fig. 3). These secondary structure elements form the core of the two lobes of a typical kinase-like fold protein [52]. Three alternative secondary structure prediction methods, Jpred, PsiPred and SOPMA, produced very similar results when applied to SELO proteins from humans, yeast and bacteria (see Fig. S2). These predictions are in agreement with the kinase domain secondary structure topology. Within the SELO alignment, there are some conserved insertions, e.g. between SELO and SELO2 groups, and in the fungal sequences, as compared to the other SELO proteins. These usually occur outside the predicted secondary structure elements.

Out of the conserved regions I–XI (subdomains), as defined by Hanks and co-workers for the first solved kinase structure, the protein kinase A (PKA) [5], the subdomains I (ATP-binding G-loop), II (lysine K72 in β-strand 3), III (glutamate 91 in α-helix C), VIb (catalytic loop), VII (DFG, Mg^2+^ -binding site), and possibly VIII (APE motif) are conserved in SELO proteins (See Fig. 3).

**Figure 4:**
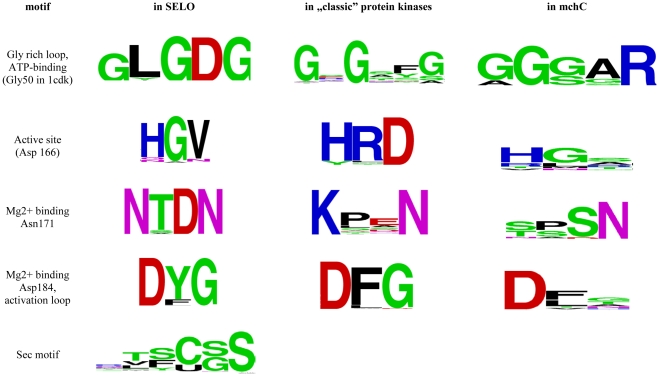
Sequence logos for the conserved motifs of the SELO family (using the 143 representative sequences), in “classic” protein kinases (Pfam family pkinase, PF00069), and in the mchC group.

Among the functionally-critical conserved kinase residues, the Lys72 (PKA numbering), involved in binding of the α and β phosphate groups of the ATP molecule [53], [54], [55] is almost invariant in the SELO family. Also, the Glu91 (binding to Lys72 by a salt bridge and stabilising it) is invariant. The archetypic HRD/YRD motif of protein kinases is not conserved in the SELO family. It is usually substituted by an HGV motif, also by QGN or HGS (see the logos in Fig. 4). Thus, the presumed kinase catalytic base (Asp166 in PKA) is missing from SELO proteins. However, it has been shown for other kinases, e.g. Erbb3, that the lack of Asp166 does not necessarily mean absence of kinase activity (see Discussion section) [18]. Moreover, the asparagine corresponding to Asn171 of PKA is very well-conserved, and is responsible for binding the second (“inhibitory”) Mg^2+^ ion. The ion-binding motif D[Y/F]G is strictly conserved, binding the first (“activating”) Mg^2+^ ion, and corresponding to Asp184 in PKA. The activation loop region contains two partly conserved tyrosine residues that may be the primary phosphorylation sites (see Fig. 3).

Finally, the network of hydrophobic residues that span the kinase fold molecule and participate in regulation of the enzymatic activity is conserved in the SELO family (see Fig. 3). This network was recently defined by Taylor and co-workers and was termed as regulatory and catalytic spines [56]. In SELO, precise identities of residues participating in the two spines may be uncertain; however, the candidate residues are conserved and can be aligned to their counterparts in typical kinases. The kinase-like fold proteins are known to vary in their structures around the typical “classic” arrangement [8]. Also, the SELO regions outside the predicted kinase domain (approx. 120 residues at the N-terminus and approx. 200 residues at the C-terminus) may fold over and augment the kinase domain.

In cases of remote sequence similarity, building a structural model is of an illustrative nature, yet it also serves as a feasibility check for the predicted structure. Here, we discuss a structural model of the SELO kinase domain (residues 150–485) built by employing the archetypical kinase structure, PKA, as a template. The ATP-binding pocket of the modelled structure of SELO allows ligand binding in a manner considerably similar to that in the classical protein kinases (PKL). The catalytic core of the protein kinases contains a nucleotide binding motif unique among proteins with nucleotide binding site [54], [55], [57]. This conserved motif, protein kinase consensus sequence GxGxxGxV, forms the secondary structure motif β-strand-turn-β-strand, making a flap over the nucleotide. Highly conserved glycine residues are crucial for stabilizing the β-strand. In the human SELO, these three glycine residues are conserved, but in a slightly different motif: GxxGxG. This motif is reminiscent of the Walker type A motif of ATP-binding proteins, which supports the role of SELO in binding ATP. In the structural model, the glycine-rich loop lies parallel to the ATP, covering it and forming hydrogen bonds to the phosphate groups of the ligand. Although the target/template alignment for SELO and PKA is not unique/reliable in all regions, many ATP–protein interactions are reproduced in the model. The conserved lysine (Lys 72 in PKA) can form an H-bond to a β phosphoryl group in the model. Other interactions conserved in the model include the hydrogen bond between N6 of purine ring and the backbone carbonyl of Glu121 of PKA, substituted by the H-bond to Asp285 in SELO, and also the hydrogen bond between N1 and the backbone amide of Val123 (PKA) that has its counterpart in a bond to Val 287 in SELO. The ribose hydroxyl group in the model is bound via the hydrogen bond formed by the side chain of Asp338 in SELO and 3′OH group of the ribose moiety (this interaction corresponds to the bond formed by Glu170 in the template structure). Also, a number of SELO residues come within 3–4 Å to provide van der Waals interactions with this part of the nucleotide.

**Figure 5:**
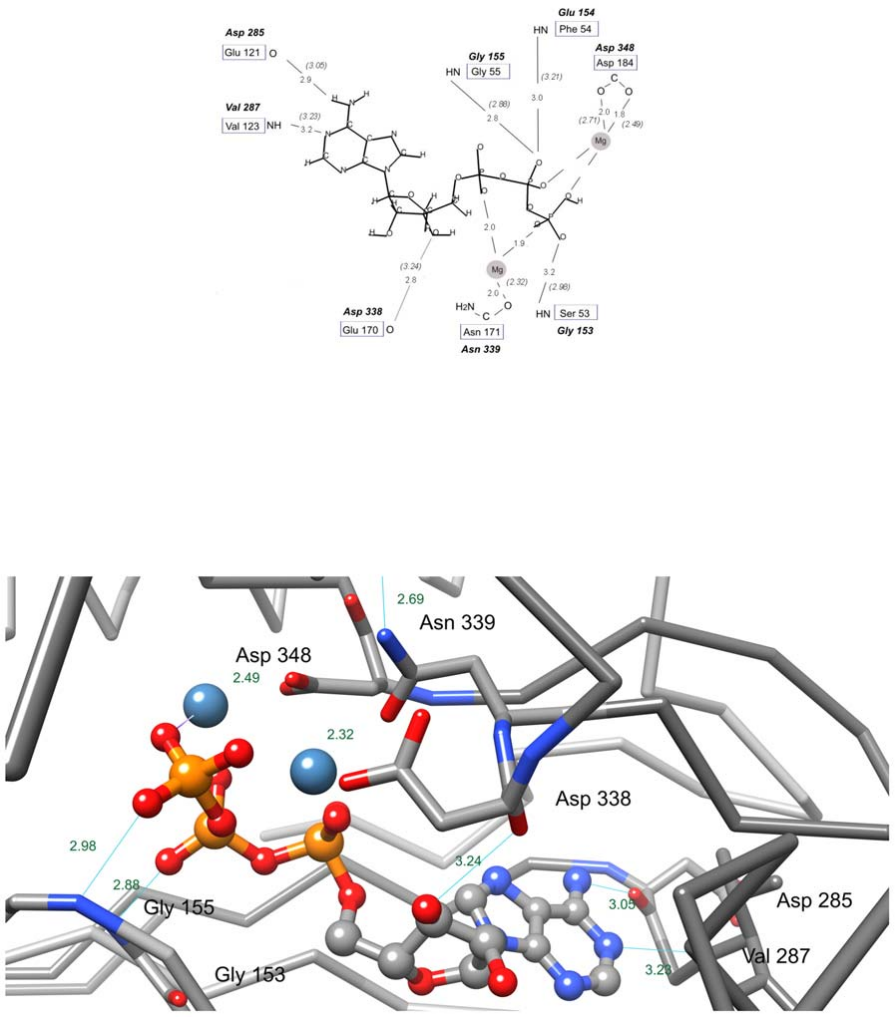
Active site details in the model of the kinase domain of the human SELO protein. *Top*. Schematic representation. Comparison of binding of an ATP molecule and two Mg^2+^ ions in SELO model (upper residue labels) and in the PKA structure (lower labels). *Bottom*. As in top panel, wireframe model of the predicted active site of human SELO with ATP and two Mg^2+^ ions.

The two residues coordinating metal ions in PKA structure are also conserved in the model: Asn171 (Asn 339 in SELO) and Asp 184 (Asp 348 in SELO). Asp 184 coordinates an Mg^2+^ ion at M2 position, which binds the metal ion more strongly in comparison to the M1 site. Binding one metal ion is essential for kinase activity; at higher magnesium concentrations a second ion binds and reduces the activity by 5-fold [58]. The metal ion coordinated by Asp184 carboxyl and phosphate β and γ groups has been postulated to be the high affinity, activating ion [59]. In the modelled SELO structure, the two Mg^2+^ ions are liganded by the carboxyl group of Asp 348 and the carboxamide group of Asn 339, the two residues conserved in the SELO family, with remaining ligands of the metal ions being provided by the phosphate groups (See Fig. 5).

The presumed catalytic base Asp166 (PKA) is substituted in SELO by Val 334 (Fig. 3) and forms a backbone-side chain hydrogen bond with the invariant Asn 339 (aligned with Asn 171 in PKA). The necessity of Asp166 for the catalysis will be discussed in the Discussion section. The second residue “suspected” of catalytic function in protein kinase CDK1 is the invariant Asn171. The amide side chain of Asn171 interacts with carbonyl oxygen of Asp166. An analogous bond is present in the SELO model, between Asn 339 and Val 334. Overall, in spite of rather far homology between SELO and porcine PKA, conservation of interactions between the ATP and protein suggests that SELO can accommodate typical kinase mode of ATP-binding and catalysis (see Fig. 5).

On the basis of the binding mode of the inhibitor bound to the PKA structure (1cdk), one may speculate about the hypothetical substrate-binding region of the SELO kinase domain. Among the residues that might participate in substrate binding if SELO bound its substrate in a way similar to PKA, one can note Leu152 (from GxxGxG glycine-rich loop), and Gly 353 (from the conserved motif FGFL in the sheet β-8 in SELO) and Glu 400 (from another conserved motif, LPLE in the helix α-F). Also, the Phe residue from the TPF motif next to the β-3 sheet in SELO could make contact with a peptide substrate. Furthermore, the motifs FYPE next to the helix α-D, and FGFLDRY situated next to the β-8 sheet may be involved in binding a presumed peptide substrate. In the latter motif, the two Phe and one leucine residue may engage in hydrophobic interactions with the substrate, while the aspartate may form hydrogen bond to a hydrophilic residue.

### Expression data on SELO genes

In the tissue expression atlas, BiopGPS (previously known as Symatlas) [60], in both human and murine normal tissues, elevated expression in liver is striking (2 and 10 times the median tissue expression, respectively in the two species).

No striking expression changes in disease were identified for human *SELO* gene in the GeneChaser or Gene Expression Omnibus databases.

In yeast, the expression of *SELO* homologue, the *FMP40* gene, is upregulated upon peroxide treatment [61]. It is also upregulated in aerobic milieu versus anaerobic one in cultures with growth limited by nutrient availability [62]. Lastly, desiccation and rehydration stress led to significant upregulation of *FMP40* [63].

For *ydiU*, the bacterial homologue of *SELO*, similar trends can be observed. Examination of the EcoCyc database [64] indicates possible *ydiU* expression differences in aerobic conditions versus anaerobic ones. Indeed, closer examination of several datasets shows statistically significant upregulation. For example, *ydiU* expression is upregulated at least two-fold under aerobic conditions as compared to anaerobic in wild-type *E. coli* and in knockout mutants lacking different stress-response sensors/response master regulators (e.g. *fnr, appY, OxyR, SoxS*) [65], suggesting *ydiU* may function in a stress-response pathway independent of well-known regulators. In a time-course experiment on transition from anaerobic to aerobic conditions, *ydiU* is upregulated 3.8-fold after 60 minutes but not at earlier time-points [66]. Also, other stress factors (e.g. pressure and temperature changes) and modulation of stress response regulators (e.g. *IscR, oxyR*) cause alteration in *ydiU* expression [67], [68].

### Genomic neighbourhoods of SELO proteins, characteristics of organisms possessing SELO genes

Lifestyle analysis of microbes harbouring *SELO* genes reveals a significant overrepresentation of aquatic organisms (as compared to a random sample of organisms). Binomial test probability of a number of aquatic species equal-or-higher than the observed number (47%) is 2.3E-11 with the expected (background) frequency of aquatic species among the organisms with known genomes being 16%. Also, there is a significant overrepresentation of aerobic organisms (observed 65%, expected 32%, p-value 4.7E-07). No significant deviations from the expected frequencies were noted for organism motility nor preferred temperature range. The enrichments for aquatic and aerobic lifestyles are independent, as seen by Fischer's exact test that estimates the p-value of the observed aquatic and aerobic lifestyle contingency table at 0.14.

Statistical analysis of genomic neighbourhoods (see Table 2) identified gene families, as defined by the COG classification system [29], significantly overrepresented in a 22 kbp genomic window centered around microbial *SELO* homologues in the MicrobesOnline system. Most notable were the genes homologous to *btuD* and *btuC* [69], [70], [71], and the ATPase and permease components of the ABC-type cobalamin transport system (p-values below 1E-10, see Methods section). Also, two COG groups related to oxidative stress response stood out: *msrB* (methionine sulfoxide reductase) and *btuE* (glutathione peroxidase) [72], [73], p-values<1E-9. Other notable gene groups, possibly related to stress response were *BaeS* (signal transduction histidine kinase, p-value below 1E-10), and *Fur* (Fe^2+^/Zn^2+^ uptake regulation proteins involved in ROS defense [74]).

**Table 2:** Functional families (COGs) overrepresented in genomic neighbourhoods of microbial SELO homologues.

COG group	Gene	COG description	Probability of finding a COG in *SELO/ydiU* neighbourhood	Probability of finding a COG in *SELO/ydiU* neighbourhood, corrected	Structure type (Pfam clan)
COG4138	*btuD*	ABC-type cobalamin transport system, ATPase component	<10^−10^	<10^−10^	AAA ATPase, CL0023
COG4139	*btuC*	ABC-type cobalamin transport system, permease component	<10^−10^	<10^−10^	Membrane transporter, CL0142
COG1957	*URH1*	Inosine-uridine nucleoside N-ribohydrolase	<10^−10^	<10^−10^	
COG0642	*BaeS*	Signal transduction histidine kinase	<10^−10^	<10^−10^	His_Kinase_A, CL0025
COG0229	*msrB*	Peptide methionine sulfoxide reductase	<10^−10^	5.90E-10	
COG0386	*btuE*	Glutathione peroxidase	<10^−10^	7.91E-10	Thioredoxin, CL0172
COG1806	*ydiA*	Uncharacterized protein conserved in bacteria. Predicted AAA ATPase	<10^−10^	2.44E-09	AAA ATPase, CL0023 (remote similarity, detected by FFAS)
COG0722	*aroF aroG aroH*	3-deoxy-D-arabino-heptulosonate 7-phosphate (DAHP) synthase	<10^−10^	3.13E-09	DAHP synthetase I, TIM_barrel, CL0036
COG0271	*BolA*	Stress-induced morphogen (activity unknown)	1.72E-10	8.39E-07	
COG2917	*yciB*	Intracellular septation protein A	1.49E-08	7.27E-05	
COG0791	*nlpC spr yafL ydhO*	Cell wall-associated hydrolases (invasion-associated proteins)	2.39E-08	0.00012	Peptidase_CA, CL0125
COG0574	*ppsA*	Phosphoenolpyruvate synthase/pyruvate phosphate dikinase	1.21E-07	0.00059	Multidomain, contains Pyruvate kinase-like TIM barrel, CL0151
COG0735	*Fur*	Fe2+/Zn2+ uptake regulation proteins	3.40E-07	0.0017	Helix-turn-helix clan, CL0123
COG4181	*SalX ybbA*	Predicted ABC-type transport system, ATPase component	3.64E-07	0.0018	AAA ATPase, CL0023

Statistical analysis using 22-kbp wide genomic windows.

Finally, for some oxidative stress response-related gene families, only uncorrected p-values indicated overrepresentation, suggesting at least a trend (correcting for multiple testing is discussed in the Methods section). These were *msrA* (peptide methionine sulfoxide reductase), and *Gst* (glutathione transferase). The *msrA* and *msrB* genes, coding for two different types of methionine sulfoxide reductases, often co-occurring with *SELO* homologues, have been shown to have an important role in ROS defense [75], [76], [77]. In addition, another group of genes encoding signalling proteins was detected: Rtn, EAL domain-containing, involved in the turnover of the second messenger, cyclic di-GMP and linking the sensing of specific environmental cues to appropriate alterations in bacterial physiology and/or gene expression [78], [79].

It is noteworthy that this type of analysis might be biased by the uneven sampling of microbial genomes sequenced to date. However, in our genomic neighbourhood analysis, only nine genera provided three or more genomes (up to seven), and these genera belonged to various main bacterial phyla: Firmicutes, Actinobacteria, Cyanobacteria, alpha- and gamma-proteobacteria, and Enterobacteria.

Together, striking among the gene groups overrepresented in the SELO genomic neighbourhoods are oxidative stress response-related genes and components of transport/efflux systems. Among the latter, there were several COG groups coding for proteins containing ABC transporter ATPase domains (Pfam family ABC_tran, PF00005): *btuD*, *salX*, *uup*, *ydiA*, and COG groups coding for Major Facilitator Superfamily transporters (Pfam clan CL0015): *araJ*, *emrA*. Also, the COG family *CirA*, outer membrane receptor proteins, involved mostly in Fe transport, was overrepresented. Although ATPase domains of the P-loop NTPase type, including the distantly related ABC and AAA+ type domains, are ubiquitous and occur in different proteins that carry out diverse functions [80], [81], the ATPase domains coded often in *ydiU* neighbourhoods belong to families present in ABC transporters.

Many *SELO*-possessing species have their taxon-specific *SELO* genomic neighbours. *Escherichia coli*, *Shigella* and *Salmonella SELO* genes are surrounded both by ABC transporter component genes and genes involved in various aspects of “basic metabolism” (phosphoenolpyruvate synthase (EC 2.7.9.2), 2-keto-3-deoxy-D-arabino-heptulosonate-7-phosphate synthase I alpha (EC 2.5.1.54) or long-chain-fatty-acid–CoA ligase (EC 6.2.1.3)). The *SELO* gene in *Francisella* co-occurs with proton/sodium glutamate and sugar symporters. *Vibrio cholerae SELO* co-occurs with genes involved in glycerophospholipid metabolism (CDP-diacylglycerol–glycerol-3-phosphate 3-phosphatidyltransferase (EC 2.7.8.5), phosphatidate cytidylyltransferase (EC 2.7.7.41), 1-acyl-sn-glycerol-3-phosphate acyltransferase (EC 2.3.1.51)) on one side and with the TRAP-type C4-dicarboxylate transport system genes on the other side. The *SELO* from *Yersinia* is a neighbour of polymyxin resistance genes and the “basic metabolism” set of genes. The *SELO* in *Bacilli* usually directly co-occurs with a proximal bifunctional protein such as zinc-containing alcohol dehydrogenase; quinone oxidoreductase (NADPH:quinone reductase) (EC 1.1.1.-) involved in glutathione-regulated potassium-efflux system which is preceded by an *ArsR* transcription factor and on the distal side with a response regulator. Not too far away there is a frequent gene encoding the GlcD subunit of the glycolate oxidase which catalyzes the first step in the utilisation of glycolate as the sole source of carbon [82].

Furthermore, analysis of the promoter region of the *ydiU* gene of *E. coli* K-12 substr. MG1655 revealed that it has a cis element that binds to the IscR transcription factor [83]. IscR, the “Iron-sulphur cluster Regulator”, is negatively autoregulated, and contains an iron-sulphur cluster that could act as a sensor of iron-sulphur cluster assembly [84], [85]. This protein regulates the expression of the *isc* and *suf* operons that encode components of pathways of iron-sulphur cluster assembly, iron-sulphur proteins, anaerobic respiration enzymes, and biofilm formation proteins [84], [85], [86], [87], [88], [89]. This is yet another piece of evidence supporting the hypothesis of role of *ydiU* in oxidative stress response.

### mchC, the microcin maturation proteins, as remote homologues of the SELO proteins

Search for distant homologues of a SELO protein, starting from a protein from *Marinomonas* sp. MED121, gi:86163056, brings up, within the second PSI-BLAST iteration, significant similarity to a group of mchC proteins present in several *Escherichia coli* strains, *Cellvibrio japonicus* Ueda107, *Klebsiella pneumoniae* RYC492, *Photorhabdus asymbiotica*, few *Vibrio* species, including two *Vibrio cholerae* strains (MZO-2 and AM-19226), and a few *Xanthomonas* species (for a list, see Table S2). The HHpred and FFAS searches on the Pfam database confirm similarity of mchC proteins to the SELO family and remote similarity to kinase families (see Table S1).

The group of about 20 mchC proteins exhibits large variability (down to 26–35% sequence identity within the group, see Figs. S4 and S5), and large differences are observed even between several mchC proteins from a single strain, *Cellvibrio japonicus* Ueda107 (down to 28% sequence identity). The characteristic kinase motifs, identified for “typical” SELO proteins are noticeable in the mchC subgroup, with the notable large variation in the motif corresponding to the kinase HGD motif and HGV motif present in most SELO proteins (see Fig. 4). The remote relation of the mchC to other SELO proteins is exemplified by the low similarity between *E. coli* ydiU and mchC proteins. The similarity is not detectable by a BLAST comparison, yet it is obvious when profile-profile methods are used, e.g. the FFAS Z-score is −88.0, and sequence identity for the 529 residue-long FFAS alignment is only 12%.

In *E. coli* strains, the *mchC* gene is a part of the *mch* gene cluster involved in production, maturation, and export of the bactericidal antibiotic microcin H47 (the corresponding gene cluster in *K. pneumoniae* is *mce*, and the appropriate microcin is termed as E492 [90], [91]. The microcins themselves are coded by a gene in the *mch* (or *mce*) cluster, and are posttranslationally modified. The mchC protein is necessary for the modification involving the covalent attachment of enterobactin, a siderophore moiety [91]. The *K pneumoniae* orthologue of mchC, mceJ, functions in a protein complex (mceI/mceJ) [92]. An analogous pair, mchC/mchD, exists in the few *E. coli* strains and some other bacteria (see Table S2). The mchD and mceI proteins are acyltransferases of hemolysin C-like structure (RTX toxin acyltransferase family, HlyC, PF02794 [93]). Since hlyC proteins are found only in bacteria, an interaction similar to mceI/mceJ cannot be expected to occur for eukaryotic SELO proteins.

Although no close homologue of mchD is annotated in the in *V. cholerae* strains MZO-2 and AM-19226 that possess mchC homologues, a tblastn search in *Vibrio* genomic sequences yields MZO-2 and AM-19226 regions of significant similarity (after translation) to the *E. coli* mchD sequence (E-value 1e-05, 30% sequence identity), just next to the regions encoding the *mchC* gene, suggesting the presence of an unannotated *mchD* homologue in the two *Vibrio* strains. Similar putative *mchD* genes can also be found in a few strains of *Xanthomonas*. This leads to a hypothesis that all mchC proteins may act as accessory proteins/regulators for the hlyC-like acyltransferases, mchD.

Interestingly, in two bacterial species that have many strains with sequenced genomes, *E. coli* and *V. cholerae*, *mchC* gene is present only in a number of strains out of dozens sequenced ones. The *E. coli* strains harbouring mchC proteins are either commensal, asymptomatic ones: bacteriuria strain 83972 [94], commensal probiotic strain Nissle 1917 [95] or pathogenic ones: strain CFT073, causing persistent diarrhea, and strain 042, causing urinary tract infections [96]. In *E. coli*, presence of the *mchC* gene coincides with presence of the whole microcin H47 gene cluster.

In *V. cholerae*, mchC homologues are found in just two strains, MZO-2 and AM-19226. These are clinical strains isolated in Bangladesh, yet different from the most deadly pandemic strains. AM-19226 is a pathogenic strain that lacks the typical virulence factors [97]. Interestingly, although as outlined above, a putative region coding for mchD can be found in these two strains, together with some other putative microcin export proteins (see below), a region of homology to the *mchB* gene that codes for the microcin H47 itself cannot be found in *Vibrio*. This does not rule out the possibility that the *mch* homology region in *Vibrio* functions toward the production, maturation and export of a yet-undiscovered H47-like microcin. Indeed, similarity is not detectable by BLAST between the well-studied and probably homologous microcins H47 and E492 from *E. coli* and *K. pneumoniae*. However, their similarity is detected by FFAS with a Zscore of borderline significance, −6.15, and sequence identity of 31%.

Other *mchC*-possessing strains are usually pathogenic. *Photorhabdus asymbiotica* [98] is an entomopathogenic bacterium, living in a mutualistic association with entomopathogenic nematodes, yet it causes wound infection in humans. It possesses a complete *mch* gene cluster, significantly similar to that of *E. coli*, including the *mchC* and microcin H47 genes. In *K. pneumoniae*, the *mce* gene cluster, homologous to the *mch* cluster, is found only in the clinically isolated strain RYC492 [99], but not in other *K. pneumoniae* strains. The mchC protein is also found in a saprotrophic soil bacterium *Cellvibrio japonicus* [100] (five distinct genes) and several strains of various species of the plant pathogen *Xanthomonas* [101], [102].

In the strain *E. coli* Nissle 1917, the *mch* cluster is found in the Genomic Island GEI I [95], which corresponds to the pathogenicity island PAI-CFT073-SerX in the strain CFT073 [96], [103]. The PAI-CFT073-serX island is also found in the strain ABU 83972 [104]. In *V. cholerae* strains MZO-2 and AM-19226, *mchC* is encoded within the genomic island GI-52, together with five other genes, including type I secretion membrane fusion protein, hlyD-like, and ABC-type bacteriocin/lantibiotic exporter, HlyB-like [105]. Interestingly, the island GI-52 has a cassette-like property, i.e. different islands occupy the same region in different strains [105]. It has to be noted that the islands containing the *mch* genes need not be virulence/pathogenicity determinants per se, but they may also confer general fitness advantage [104]. Yet, it has been reported that particular microcins, including the *mch* cluster encoded ones, corresponded to specific urovirulence gene profiles [106]. Also, there is an overrepresentation of *E. coli* urinary tract infection strains among those encoding microcin H47 [107].

## Discussion

We present the discovery of a novel kinase-like family with a member in humans and presence throughout the tree of life as a small step towards filling in the blank spots in the complex regulatory machinery of the living cell. Precise charting of the human kinome is important for several reasons. The recent timely essay “Too many roads not taken” [108] convincingly presents the case for exploring the “unpopular“ members of otherwise popular protein families. The authors point out that even for the established and attractive drug targets, kinases, majority of scientific activity, including articles and patents, focuses on a minority of proteins that have been historically popular. The “uncharacterised” proteins [19] are even less fortunate, often getting little attention even if the experimental data point at their involvement in interesting biological or disease processes [109].

Also, many kinase inhibitors targeted at well-known kinases may have off-target effects onto the less-studied ones [110]. It has been reported that some kinases affected by off-target effects of a compound have less than 20% sequence identity in their active sites as compared to the original target kinase [110]. Thus, complete understanding of the kinome is important for proper assessment of the potential off-target effects of kinase-modulating compounds.

Although the kinomes are remarkably well-studied, it is predicted that they may be not fully mapped yet, even in the well-studied organisms. Novel protein kinases are expected in bacteria, e.g. the only two annotated protein kinases in *Mycoplasma pneumoniae* account for only five out of 63 identified protein phosphorylation events [111].

The correlation of presence of *SELO* genes in bacteria with aquatic and aerobic lifestyles aligns with the hypothesis that these genes are involved in stress responses, possibly in oxidative stress response. It has been shown that genes involved in cellular responses to oxygen occur much more often among aerobes than in anaerobes [112]. This is corroborated by our genomic neighbourhood analyses. Although *SELO* genes in bacterial genomes are located in clusters of diverse functions, a recurring functional theme involves transport and oxidoreductase functions. The transport and stress response roles are not excluding, since export of redox-cycling antibiotics is one of the mechanisms of defense against oxidative stress [113]. These findings are further supported by the observation of the most prominent expression differences measured for *SELO* genes that involve stress factors, such as aerobic conditions versus anaerobic. Lastly, the predicted kinase function, together with the frequent but not invariant cysteine- or selenocysteine-containing Cxx[CU] motif, suggestive of an oxidoreductase function, indicate a possibility of SELO proteins' involvement in kinase signalling coupled to redox detection/signalling. Since no fold prediction has been possible for the selenocysteine-containing C-terminal region, no ultimate function assignment can be done. However, several lines of circumstantial evidence, including genomic neighbourhoods, gene expression data, microbial lifestyles and presence of Cys-Sec motif, point toward the involvement of SELO in response to oxidative stress. The C-terminal Cxx[CU] motif, although not always present throughout the SELO family, occurs in all major lineage representatives, which supports the hypothesis of its functional relevance.

In the *mchC* branch of the *SELO* family, mchC functions in maturation of molecules secreted via a hemolysin-like type 1 secretion system [114]. There is an obvious similarity between the secretion system coded in the *mch* gene cluster, and the genomic neighbourhoods of *ydiU*-like genes. For example, mchF protein serves a similar role in the btuC/btuD pair [115] [69], coded by genes frequent in *ydiU* neighbourhoods, with homologous ATPase domains (btuD protein and C-terminal part of the mchF protein) and structurally different membrane-spanning regions (btuC protein and central part of mchF). This suggests that both ydiU-like and mchC-like bacterial proteins as well as their eukaryotic homologues may be involved in the regulation of secretion or transport processes.

The predicted mitochondrial localisation of metazoan SELO proteins and the experimentally detected presence of SELO homologues in yeast mitochondria are consistent with the known antioxidant activities of mitochondrial kinases [116], [117].

Can the kinase function prediction for SELO proteins be trusted, or is it only a reliable three-dimensional fold prediction? According to previous studies on protein kinase catalysis, phosphoryl transfer requires a basic residue to accept the proton from the attacking hydroxyl group [118], [119] and the main candidate for this key residue absolutely required for kinase activity, and with sufficient p*K*_a_ in negatively charged environment was Asp166 of PKA, which is not conserved in SELO proteins. Asp166 is one of the most conserved amino acid residues found in all PKL kinases [6]. However, for the human Erbb3 protein, having Asn at a position corresponding to Asp166 and long believed to be a pseudokinase, Lemmon and colleagues have recently demonstrated some residual kinase activity [18]. They proposed an atypical catalytic mechanism whereby the attacking proton from the tyrosyl substrate moves to γ- and then β-phosphate group of the ATP molecule instead of the aspartate corresponding to D166 in PKA. Similar results were previously shown by mutating the residue corresponding to Asp166 in EGFR (Asp to Ala change) [120] and showing retained biological function. Another example of atypical active kinases are the WNK (with no K) kinases, lacking the ultra-conserved Lys72 [121] whereas a lysine located elsewhere in the sequence assumes the role of Lys72. Also, recent work by Manning and co-workers [6] provides evidence of rich diversity of kinase families in the marine metagenome, with several families lacking one or more key catalytic motifs previously identified in studies on eukaryotic PKL kinases.

Therefore, as summarised recently by Taylor, “It is difficult to say unambiguously given kinase will be inactive” [122]. Manning and colleagues have performed structure comparison of an inactive pseudokinase and a closely homologous active kinase counterpart [123]. They have discussed various alterations in the conserved kinase motifs and concluded that provided the ATP-binding G-loop is functional, other conserved motifs may be at times compensated for, if missing or altered.

For very distantly related homologues, the sequence alignment details are known to be less reliable than the overall detection of homology stemming from significant similarity [124]. Thus, some of our definitions of SELO kinase active site short motifs, e.g. the location of the classic Lys 72, may be erroneous. Also, even if the kinase function predicted for SELO proteins is true, it is not straightforward to predict the substrate. Among the top-ranking hits for SELO, there were both lipopolysaccharide kinases [27] and protein kinases. However, the lipopolysaccharide kinase prediction seems less likely, because this family lacks the DFG/DYG motif responsible for the magnesium ion binding in the catalytic site. This motif is strictly conserved among protein kinases, as well as among SELO homologues.

It has been previously demonstrated that many free-living marine microbes possess homologues of virulence genes, hence they propose a hypothesis that disease – related genes may be sometimes originating from marine bacteria invading eukaryotic hosts in the marine environment [125]. This adds to the appeal of studying protein families with atypical phylogenetic distribution, such as the SELO/ydiU family.

The challenge of turning a structure prediction into a useful function prediction [126] involves specifying particular suggestions for experimental validation. It is even more challenging to turn the molecular function prediction into a biological process prediction, the latter usually being not directly linked to protein structure. Here, we strived to achieve this, complementing structural predictions with the additional analyses of available functional data, e.g. phylogeny, microbial habitat and lifestyle, and gene expression data. The ultimate answers, nevertheless, will only come from experiments, both biochemical and biological. Some of the approaches aimed at validation of the SELO kinase function may include standard assays for ATP binding and hydrolysis using ATP derivatives [127] and comparing SELO proteins with mutants having putative active site disrupted. Other approaches may include proteomics analyses of overall protein phosphorylation changes in cell systems [128] upon disruption of the predicted SELO kinase fingerprint. Furthermore, other cell phenotypic features, such as resistance to oxidative stress may be monitored upon modulation of SELO protein expression.

## Methods

### Identification of the SELO domain, structure prediction, sequence analysis

For remote homology identification, PSI-BLAST searches were executed using the standard parameters on the nr and env_nr databases at NCBI as of June 2010. Saturated BLAST [30] searches used five iterations of PSI-BLAST on nr and env_nr databases, BLAST expect value of 0.001 and redundancy threshold for selection of representative sequences set to 60% identity as criteria for seed selection. Also, HHsenser [26] was used, querying the nr and env_nr sequence databases, with standard parameters. For Pfam domain assignments, HMMER3 [129] on the Pfam [31] database as of January 2011 were used.

For survey of similarities within the thioredoxin-like clan, the CLANS algorithm [34] was run on a set of sequences including (a) all the Pfam “seeds” from the 17 families of the “protein kinase domain” clan (CL0016), (b) the 143 representative SELO domains (see Results) along with 10 representative mchC proteins, (c) other proteins with expected similarity to the PKL clan, including all the Pfam “seeds” from the Pfam families: Alpha_kinase (PF02816) [36], PI3_PI4_kinase (PF00454) [130], Act-Frag_cataly kinase, PF09192, [131], PPDK_N (PF01326) [132], PIP5K (PF01504) [133]. For these families, structural similarity to the PKL kinases is known [8]. CLANS was run with five iterations of PSI-BLAST, using the BLOSUM45 substitution matrix and inclusion threshold of 0.001. For the graph, similarity relations with significance of P-value below 0.1 were considered.

Transmembrane region predictions were achieved by the TMHMM and MEMSAT servers [134], [135]. The Jpred, PsiPred and SOPMA servers were used to predict the secondary structure of the SELO domains [136], [137], [138]. Multiple alignments of the SELO domain were constructed by using the PROMALS [139] and MUSCLE programs [140]. The former includes predicted secondary structures in the buildup of the alignment, and both employ remote homologues of the aligned sequences. For the multiple alignments, only regions that could be aligned to the central part of the human SELO protein (region 150 to 450) were used. Prediction of subcellular localisation was performed using TargetP and MultiLoc [141], [142].

For three-dimensional structure prediction, two methods were used, namely FFAS03 [25] that uses sequence profile-to-profile comparison and HHpred [26] that employs HMM-to-HMM comparison. Both methods queried the Pfam, PDB and SCOP [143] databases.

Phylogenetic analyses were performed using the phylogeny.fr server [144], employing the maximum likelihood method PhyML, with the Approximate Likelihood-Ratio Test (aLRT) for branch support estimation. The tree was constructed using MUSCLE multiple sequence alignment for the set of 143 representative SELO domains and visualised using the iTOL tool [145]. The sequence variability was displayed as sequence logos using the WebLogo server [146].

Since selenocysteine is often misannotated as a stop codon, for the representative set of 143 SELO proteins, we examined a multiple alignment of the C termini of these proteins, and were able to detect the likely misannotation of Sec residues at position Uxx> in the following six proteins: NCBI gi:145355852 [*Ostreococcus lucimarinus*], 224011106 [*Thalassiosira pseudonana*], 260794380 [*Branchiostoma floridae*], 219126041 [*Phaeodactylum tricornutum*], 195999240 [*Trichoplax adhaerens*], 116056105 [*Ostreococcus tauri*].

### Structure model of the SELO domain

Sequence alignment between the kinase-like region of SELO and the PKA structure (PDB code:1cdk) was produced by the FFAS03 structure prediction method and manually adjusted to accommodate predicted secondary structures. Three-dimensional structure models were constructed by the program MODELLER [147]. The modelled region included the catalytic domain of human selenoprotein O (NP._113642.1), residues 150–487, and was constructed on the basis of the crystal structure of catalytic subunit of the c-AMP–dependent protein kinase from porcine heart (PDB:1cdk) which includes an ATP analogue, two Mn^2+^ ions and a peptide derived from protein kinase inhibitor. ATP analogue from the template was replaced by ATP molecule and Mn^2+^ ions were replaced by Mg^2+^ ions in the final model. The combined Modeller9v8 Python scripts (automodel mode) model-multichain and model-ligand were used. Out of the models presented by MODELLER, the one with most favourable *molpdf* score was selected for further analysis. The MetaMQAP server [148] was used to estimate the correctness of the 3D models using a number of model quality assessment methods in a meta-analysis.

### Analysis of microbial SELO domains

Habitat and lifestyle information for SELO protein-possessing microbes was collected from the Microbial Genomes resource within Entrez Genome Project database at NCBI. Analysis of genome environments of the SELO bacterial species was performed using SEED [149], [150], MicrobesOnline [151] and Integrated Microbial Genomes (IMG) [33]. Significance of enrichment of the SELO neighbourhoods in gene classes was performed as following. Genes annotated with COG database identifiers [29] were identified in SELO homologue neighbourhoods in 265 genomes in the MicrobesOnline system, within 11 kbp in both directions. Probability of finding a gene from a particular COG group in the SELO neighbourhood was estimated using the binomial test. The background probability of finding a particular COG group in any genomic region of the given size (22 kbp) was estimated using the data on the COG occurrence in 66 representative microbial genomes analysed by the COG authors [29]. The resultant probability was adjusted using the Bonferroni correction, taking into account the total number of 1003 COGs tested (all that were observed in the SELO neighbourhoods).

Gene coexpression was studied using the STRING tool [152]. Gene expression data was extracted for *SELO* genes using the BioGPS system [60], and the Genechaser and GEO databases [153], [154].

## Supporting Information

Figure S1 **SELO phylogenetic tree (PhyML) including representative sequences from all domains of life as well as the marine metagenomic sequences.** Tree branch coloring: Red: vertebrates, Yellow: non-vertebrate Metazoa, Brown: Fungi, Green: other eukaryotes including green algae and stramenopiles, Light blue: Bacteria, Dark blue: Archaea: Inner and outer rings denote the presence of Cys or Sec at C-terminus, as in Figure 2.
(PDF)

Figure S2 **Secondary structure predictions for four selected SELO proteins, in a MUSCLE Multiple sequence alignment (PsiPred** [**155**] **, Jpred** [**137**] **, Sopma** [**138**] **).** Secondary structure elements named as PKA, according to Knighton [51]. “x” denotes putative residues belonging to the C-spine (catalytic), “+” denotes putative R-spine (regulatory) residues. Exclamation signs denote potential phosphorylation sites in the activation loop. Locations of predicted key catalytic residues shown in standard PKA numbering (e.g. H166).
(DOC)

Figure S3 **Multiple sequence alignment (MUSCLE) of selected eukaryotic SELO proteins (used for construction of the tree shown in** **Fig. 2** **).** Identifiers: NCBI gi numbers.
(DOC)

Figure S4 **Multiple sequence alignment (MUSCLE) of mchC proteins, with human SELO and** ***Escherichia coli*** **ydiU added.** Identifiers: NCBI gi numbers.
(RTF)

Figure S5 **Phylogenetic tree (PhyML) of mchC proteins, with human SELO and** ***Escherichia coli*** **ydiU added, constructed using the alignment from Figure S4.** Identifiers: NCBI gi numbers.
(PDF)

Table S1 **Structure predictions for SELO proteins.** Assignments of human SELO protein (gi: 172045770), yeast (*S. cerevisiae*) FMP40 protein (gi: 3183490), *E. coli* ydiU protein (gi: 16129662) and *E. coli* mchC protein (gi: 47600579) to PDB, SCOP and Pfam using FFAS and HHpred methods. Top hits shown for each combination of method, query protein, and database. For Scop hits, d.144 denotes members of Protein kinase-like (PK-like) fold.
(DOC)

Table S2 **Bacterial strains possessing** ***mchC*** **genes.**
(DOC)
