# Supplementary figures and images for: A Novel Protein Kinase-Like Domain in a Selenoprotein, Widespread in the Tree of Life
Source: PLoS One. 2012 Feb 16;7(2):e32138. doi: 10.1371/journal.pone.0032138 (PMC3281104; doi:10.1371/journal.pone.0032138)

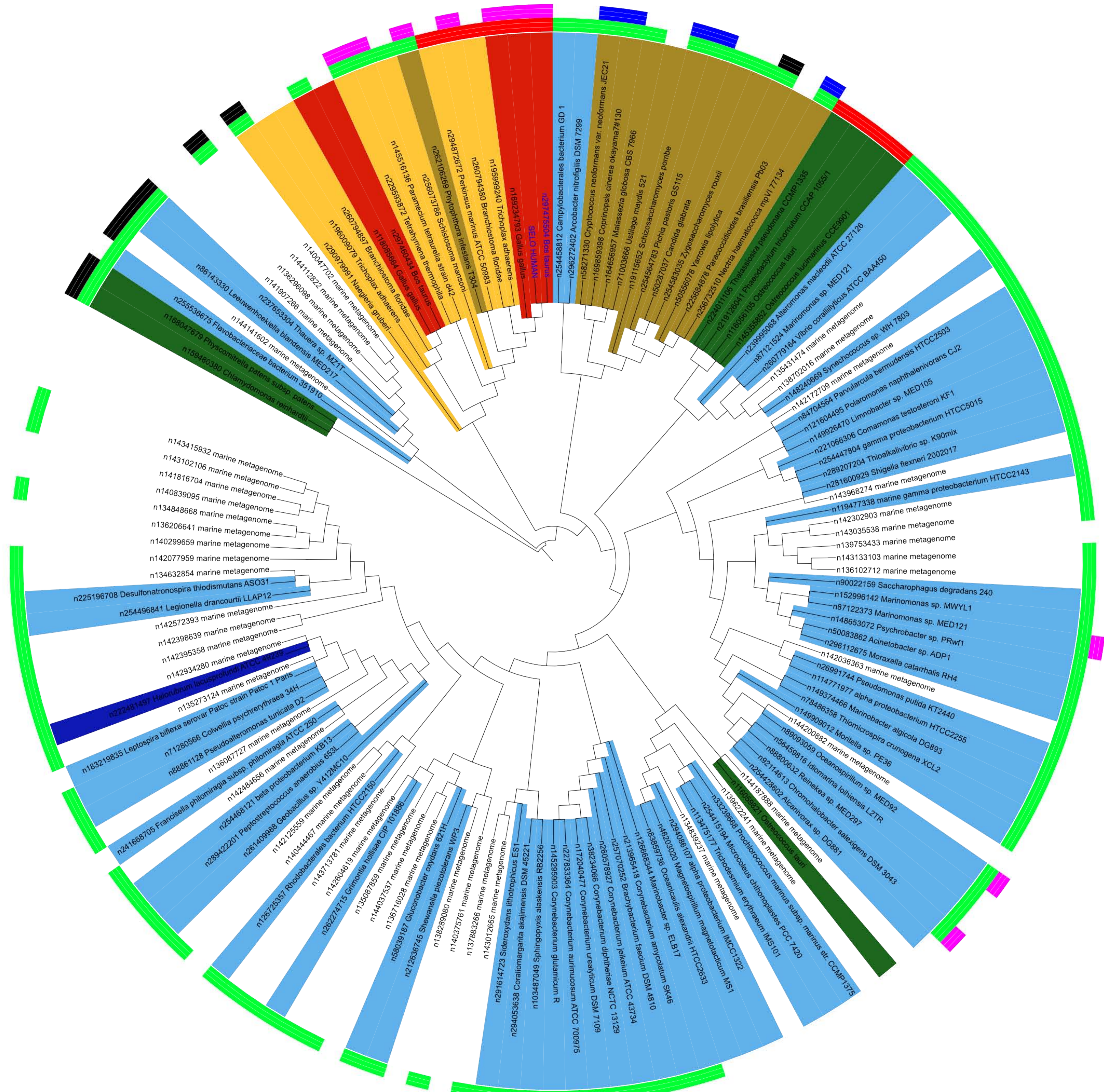

Supplement: Figure S1 — SELO phylogenetic tree (PhyML) including representative sequences from all domains of life as well as the marine metagenomic sequences. Tree branch coloring: Red: vertebrates, Yellow: non-vertebrate Metazoa, Brown: Fungi, Green: other eukaryotes including green algae and stramenopiles, Light blue: Bacteria, Dark blue: Archaea: Inner and outer rings denote the presence of Cys or Sec at C-terminus, as in Figure 2. (PDF) [file pone.0032138.s001.pdf]

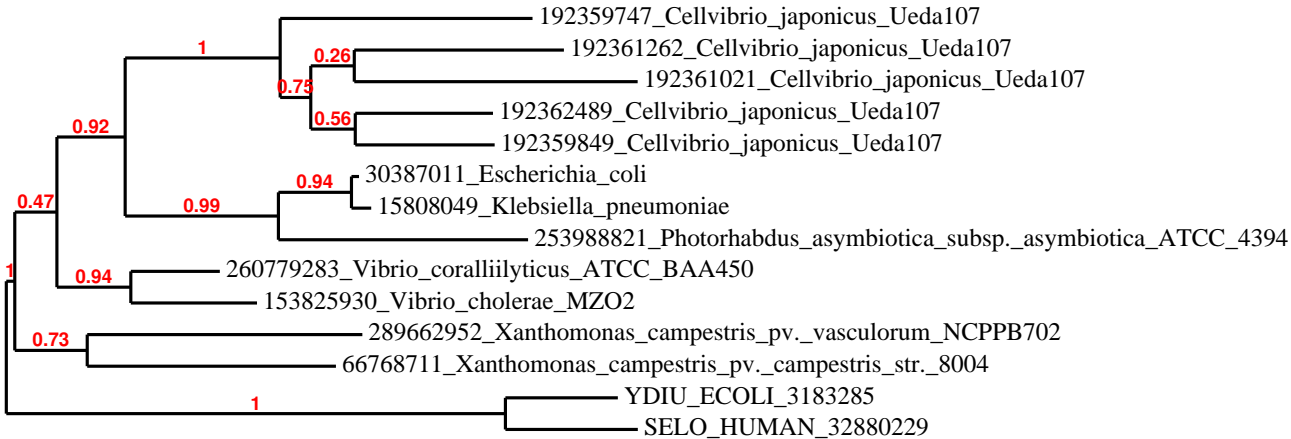

Supplement: Figure S5 — Phylogenetic tree (PhyML) of mchC proteins, with human SELO and Escherichia coli ydiU added, constructed using the alignment from Figure S4. Identifiers: NCBI gi numbers. (PDF) [file pone.0032138.s005.pdf]
