# Supplementary material for: A Novel Protein Kinase-Like Domain in a Selenoprotein, Widespread in the Tree of Life
Source: PLoS One. 2012 Feb 16;7(2):e32138. doi: 10.1371/journal.pone.0032138 (PMC3281104; doi:10.1371/journal.pone.0032138)
Supplement: Figure S2 — Secondary structure predictions for four selected SELO proteins, in a MUSCLE Multiple sequence alignment (PsiPred [155] , Jpred [137] , Sopma [138] ). Secondary structure elements named as PKA, according to Knighton [51]. “x” denotes putative residues belonging to the C-spine (catalytic), “+” denotes putative R-spine (regulatory) residues. Exclamation signs denote potential phosphorylation sites in the activation loop. Locations of predicted key catalytic residues shown in standard PKA numbering (e.g. H166). (DOC) [file pone.0032138.s002.doc]

10 20 30 40 50 60 70 80 90 100 110 120

....|....|....|....|....|....|....|....|....|....|....|....|....|....|....|....|....|....|....|....|....|....|....|...

*Fmp40p_S.cerevisiae*  AQLGDGRVVNLFDLKDKCSGQWQTFQLKGAGMTPFSRFADGKAVLRSSIREFIMSEALHSIGIPSTRAMQLTLLPGTKAQRR------NQEPCAVVCRFAPSWIRLGNFNLFRWRHD---

*sopma Fmp4*  eeccttceeeeeecccttcccceeeeettttcccccccctthhhhhhhhhhhhhhhhhhhttcccccheeeeeccccchhcc~~~~~~cccchheeeehcctheeecceeehhhccc~~~

*Jpred Fmp4*  -------EEEEEEEE-----EEEEEEE---------------E----HHHHHHHHHHHHH-------EEEEEE----EEEE-~~~~~~------EEEEE-----EEEEEEEHH----~~~

*PsiPred Fmp4*  CCCCCCCCHHHCEEECCCCCEEEEEEEECCCCCCCCCCCCCCCCHHHHHHHHHHHHHHHHCCCCCCCCEEEEECCCCCCCCC~~~~~~CCCCCCEEECCCCCCEEEECCCCCCCCCC~~~

*SELO2_B.taurus* DQLGDGRAHLIGIYMNRQGEKWE-LQLKGSGKTPYSRNGDGRAILRSSLREFLCSEAMHYLGIPTSRAASLVVSDDVVWRDQFYNGNLTKERGAVVLRVAKSWFRIGSLEILTHSGE---

*sopma BOS2*  ccccttceeeeeeecccttceee~eeettttccccccccccchhhhhhhhhhhhhhhhhhttcccccheeeeecccchhcceeccccccccttheeeehcctheeecchhhhhhhcc~~~

*Jpred_Bos2*  ------EEEEEEEEE-----EEE~EEE---------------E-----HHHHHHHHHHH--------EEEEEE----EEEEE-----------EEEEEE-----EEEEEEE------~~~

*Psipred_Bos2*  CCCCCCCHHHCEEEECCCCCEEE~EEEECCCCCCCCCCCCCCCCHHHHHHHHHHHHHHHHCCCCCCCCEEEEECCCCEEECCCCCCCCCCCCCEEEEEECCCCEEECCHHHCCCCCC~~~

*YDIU_ECOLI*  GQLGDGRGILLGEQLLADGTTMD-WHLKGAGLTPYSRMGDGRAVLRSTIRESLASEAMHYLGIPTTRALSIVTSDSPVYRE-------TAEPGAMLMRVAPSHLRFGHFEHFYYRRE---

*sopma_ydiuEC*  ccccttceeeehhhcccttccee~eeetttcccccccccccchhhhhhhhhhhhhhhhhhttcccchheeeeecccccccc~~~~~~~cccctheeeehcctteeecceeehhcccc~~~

*Jpred_ydiuEC*  -------EEEEEEEE-----EEE~EEE---------------E----HHHHHHHHHHHH--------EEEEEE---EEEE-~~~~~~~-----EEEEEE-----EEEEEEHHHH---~~~

*Psipred_ydiuEC*  CCCCCCCCHHHEEEECCCCCEEE~EEEECCCCCCCCCCCCCCCCCCHHHHHHHHHHHHHHCCCCCCCCEEEEECCCCCCCC~~~~~~~CCCCCCEEEEECCCCCEECHHHHCCCCCC~~~

*SELO_H.sapiens*  GQLGDGAAMYLGEVCTATGERWE-LQLKGAGPTPFSRQADGRKVLRSSIREFLCSEAMFHLGVPTTRAGACVTSESTVVRDVFYDGNPKYEQCTVVLRVASTFIRFGSFEIFKSADEHTG

*sopma SELO_HUM*  ccccttceeeehhhccttcceee~eeetttcccccccccccchhhhhhhhhhhhhhhhhhttccccchheeecccchhhhcccccccccccchheeeehccthceecceeehhccccccc

*Jpred_SELO_HUM*  -------EEEEEEEE-----EEE~EEE---------------E----HHHHHHHHHHHH--------EEEEEE----EEEEE----------EEEEEEE-----EEEEEEEH--------

*PsiPred_SELO_HUM*  CCCCCCCEEEEEEEECCCCCEEE~EEEECCCCCCCCCCCCCCCCCCHHHHHHHHHHHHHHCCCCCCCCCEEEECCCCEEECCCCCCCCCCCCCEEEEEEECCCEECCHHHHHHCCCCCCC

beta-2 beta-3 alpha-C beta-4 beta-5

130 140 150 160 170 180 190 200 210 220 230 240

....|....|....|....|....|....|....|....|....|....|....|....|....|....|....|....|....|....|....|....|....|....|....|...

*Fmp40p_S.cerevisiae* --------LKGLIQLSDYCIEELFAGGTQFEGKPDFNIFKRDFFPDTETKIDEQVEKDETEVSTMTGDNISTLSKYDEFFRHVVSLNANTVAHWQAYGFANGVLNTDNTSIMGLTIDYGP

*sopma Fmp4*  ~~~~~~~~hhhhhhhhhhhhhhhhhhhhhcccccchhhhhhccccccccccccccccttceeeecccccchhhhhhhhhhhhhhhhhhhhhhhhhhhteehteeccttceeeeeeecccc

*Jpred Fmp4*  ~~~~~~~~HHHHHHHHHHHHHHH--------------------------------------------------HHHHHHHHHHHHHHHHHHHHHHHHHHH---------EEEEE------

*PsiPred Fmp4*  ~~~~~~~~HHHHHHHHHHHHHHHCCCCCCCCCCCCCCCCCCCCCCCCCCHHHHHHHHHHHHHCCCCCCCCCCHHHHHHHHHHHHHHHHHHHHHHHHHHHHHCCCCCCCCCCCCCCCCCCC

*SELO2_B.taurus/191-478*  --------LDLLRMLLDFII--------------------QEYFPLVD---------------------VKEPNRYVDFFSIVVFETAQLIALWMSVGFAHGVCNTDNFSLLSITIDYGP

*sopma BOS2*  ~~~~~~~~hhhhhhhhhhhh~~~~~~~~~~~~~~~~~~~~htccchhh~~~~~~~~~~~~~~~~~~~~~hccchhhhhhhhhhhhhhhhhhhhhhhhteeeeecccttceeeeeeecccc

*Jpred_Bos2*  ~~~~~~~~HHHHHHHHHHHH~~~~~~~~~~~~~~~~~~~~HHH-----~~~~~~~~~~~~~~~~~~~~~----HHHHHHHHHHHHHHHHHHHHHHHHHHH---------EEE--------

*Psipred_Bos2*  ~~~~~~~~HHHHHHHHHHHH~~~~~~~~~~~~~~~~~~~~HHHCCCCC~~~~~~~~~~~~~~~~~~~~~CCCCCHHHHHHHHHHHHHHHHHHHHHHHHHHCCCCCCCCCCCCCEEEECCC

*YDIU_ECOLI/81-357*  --------SEKVRQLADFAI--------------------RHYWSHLA----------------------DDEDKYRLWFSDVVARTASLIAQWQTVGFAHGVMNTDNMSLLGLTLDYGP

*sopma_ydiuEC*  ~~~~~~~~hhhhhhhhhhhh~~~~~~~~~~~~~~~~~~~~htcccccc~~~~~~~~~~~~~~~~~~~~~~cccchhhhhhhhhhhhhhhhhhhhhhhteeeeeeccttceeeeeeecccc

*Jpred_ydiuEC*  ~~~~~~~~HHHHHHHHHHHH~~~~~~~~~~~~~~~~~~~~HHHH----~~~~~~~~~~~~~~~~~~~~~~----HHHHHHHHHHHHHHHHHHHHHHHHHE---E-----EEE--------

*Psipred_ydiuEC*  ~~~~~~~~HHHHHHHHHHHH~~~~~~~~~~~~~~~~~~~~HHHCCCCC~~~~~~~~~~~~~~~~~~~~~~CCCCHHHHHHHHHHHHHHHHHHHHHHHHHHCCCCCCCCCCCCCCCCCCCC

*SELO_H.sapiens/150-451*  RAGPSVGRNDIRVQLLDYVI--------------------SSFYPEIQ-----------------AAHASDSVQRNAAFFREVTRRTARMVAEWQCVGFCHGVLNTDNMSILGLTIDYGP

*sopma SELO_HUM*  ccccccchhhhhhhhhhhhh~~~~~~~~~~~~~~~~~~~~htccchhh~~~~~~~~~~~~~~~~~hcccchhhhhhhhhhhhhhhhhhhhhhhhhheeeeeeeeccttceeeeeeeeccc

*Jpred_SELO_HUM*  --------HHHHHHHHHHHH~~~~~~~~~~~~~~~~~~~~HHHHHHH-~~~~~~~~~~~~~~~~~-------HHHHHHHHHHHHHHHHHHHHHHHHHHEE---------EEEEE------

*PsiPred_SELO_HUM*  CCCCCCCCHHHHHHHHHHHH~~~~~~~~~~~~~~~~~~~~HHCCHHHH~~~~~~~~~~~~~~~~~HCCCCCHHHHHHHHHHHHHHHHHHHHHHHHHHHHHCCCCCCCCCEEEEEEECCCC

alpha-D alpha-E beta-6 beta-7

250 260 270 280 290 300 310 320 330 340 350 360

....|....|....|....|....|....|....|....|....|....|....|....|....|....|....|....|....|....|....|....|....|....|....|....|

*Fmp40p_S.cerevisiae* FAFLDKFEPSFTPNHDDTAKRYSFANQPSIIWWNLQQFAKDLACLLGPEARDLELLLKGELNSVDDALEKTMIERVQKLVELSANEYKYVFTTRYAQIMSQRLGVDLDLEKCMSSTNLKD

*sopma Fmp4*  ceeeecccttcccccccccceeeeccccceehhhhhhhhhhhhhhhccccchhhhhhhhhhhhhhhhhhhhhhhhhhhhhhhhhhhhhhhhhhhhhhhhhhhttccccchhhhhhtchhh

*Jpred Fmp4*  ----------EEE-------EE-----HHHHHHHHHHHHHHHH------HHHHHHHHHHHHHHHHHHHHHHHHHHHHHHHH------HHHHHHHHHHHHHHH--------HHHH------

*PsiPred Fmp4*  CCCCCCCCCCCCCCCCCCCCCCCCCCCHHHHHHHHHHHHHHHHHHCCCCHHHHHHHHHHHHHHHHHHHHHHHHHHHHHHHHHHHHHHHHHHHHHHHHHHHHHCCCCCCHHHHHHHCCHHH

*SELO2_B.taurus/191-478*  FGFMEAYNPDFVPNTSDDERRYKIGNQANIGMFNLNKLLQALNPLLNPRQKQL----------VTQI--------------L--KEYPVLYYTRFRELFKAKLG-------------LL-

*sopma BOS2*  ceeeccccttccccccccccceeecccccchhhhhhhhhhhhhhhhhhhhhhh~~~~~~~~~~hhhh~~~~~~~~~~~~~~h~~hhhhhhhhhhhhhhhhhhht~~~~~~~~~~~~~cc~

*Jpred_Bos2*  -E------------------EE-----HHHHHHHHHHHHHHHH------HHHH~~~~~~~~~~HHHH~~~~~~~~~~~~~~H~~HHHHHHHHHHHHHHHHHHH-~~~~~~~~~~~~~--~

*Psipred_Bos2*  CCCCCCCCCCCCCCCCCCCCCCCCCCCHHHHHHHHHHHHHHHHCCCCCHHHHH~~~~~~~~~~HHHH~~~~~~~~~~~~~~H~~HHHHHHHHHHHHHHHHHHCC~~~~~~~~~~~~~CC~

*YDIU_ECOLI/81-357*  FGFLDDYEPGFICNHSDHQGRYSFDNQPAVALWNLQRLAQTLSPFV---AVDA----------LNEA--------------L--DSYQQVLLTHYGERMRQKLG-------------FM-

*sopma_ydiuEC*  ceeeccccttcccccccttcceeeccccthhhhhhhhhhhhhhhhc~~~cccc~~~~~~~~~~hhhh~~~~~~~~~~~~~~h~~hhhhhhhhhhhhhhhhhhht~~~~~~~~~~~~~cc~

*Jpred_ydiuEC*  -E------------------EE-----HHHHHHHHHHHHHHHH---~~~-HHH~~~~~~~~~~HHHH~~~~~~~~~~~~~~H~~HHHHHHHHHHHHHHHHHHH-~~~~~~~~~~~~~--~

*Psipred_ydiuEC*  CCCCCCCCCCCCCCCCCCCCCCCCCCCHHHHHHHHHHHHHHHHHHC~~~CHHH~~~~~~~~~~HHHH~~~~~~~~~~~~~~H~~HHHHHHHHHHHHHHHHHHCC~~~~~~~~~~~~~CC~

*SELO_H.sapiens/150-451*  FGFLDRYDPDHVCNASDNTGRYAYSKQPEVCRWNLRKLAEALQPEL---PLE-----------LGEA--------------ILAEEFDAEFQRHYLQKMRRKLG-------------LVQ

*sopma SELO_HUM*  ceeeccccttccccccccccceeeccccthhhhhhhhhhhhhhhhc~~~ccc~~~~~~~~~~~hhhh~~~~~~~~~~~~~~hhhhhhhhhhhhhhhhhhhhhhe~~~~~~~~~~~~~ecc

*Jpred_SELO_HUM*  -EE-----------------EE-----HHHHHHHHHHHHHHHH---~~~--H~~~~~~~~~~~HHHH~~~~~~~~~~~~~~HHHHHHHHHHHHHHHHHHHHH--~~~~~~~~~~~~~---

*PsiPred_SELO_HUM*  CCCCCCCCCCCCCCCCCCCCCCCCCCHHHHHHHHHHHHHHHHCCCC~~~CCC~~~~~~~~~~~HHHH~~~~~~~~~~~~~~HHHHHHHHHHHHHHHHHHHHHCC~~~~~~~~~~~~~CCC

beta-8 beta-9 alpha-EF alpha-F

370 380

....|....|....|....|....|..

*Fmp40p_S.cerevisiae/129-498* IEHAAEKAKEFCDVIVEPLLDILQATK

*sopma Fmp4*  hhhhhhhhhhhhhhhhhhhhhhhhhht

*Jpred Fmp4*  -----HHHHHHHHHHHHHHHHHHHH--

*PsiPred Fmp4*  HHHHHHHHHHHHHHHHHHHHHHHHHCC

*SELO2_B.taurus/191-478*  GKSEGD------DDLIAFLLHLMEKTE

*sopma BOS2*  cccccc~~~~~~hhhhhhhhhhhhhhh

*Jpred_Bos2*  ----HH~~~~~~HHHHHHHHHHHHH--

*Psipred_Bos2*  CCCCCC~~~~~~HHHHHHHHHHHHHCC

*YDIU_ECOLI/81-357*  TEQKED------NALLNELFSLMARER

*sopma_ydiuEC*  ccccch~~~~~~hhhhhhhhhhhhhht

*Jpred_ydiuEC*  ----HH~~~~~~HHHHHHHHHHHHH--

*Psipred_ydiuEC*  CCCCCH~~~~~~HHHHHHHHHHHHHCC

*SELO_H.sapiens/150-451*  VELEED------GALVSKLLETMHLTG

*sopma SELO_HUM*  ccccch~~~~~~hhhhhhhhhhhhhtt

*Jpred_SELO_HUM*  ----HH~~~~~~HHHHHHHHHHHH---

*PsiPred_SELO_HUM*  CCCCCH~~~~~~HHHHHHHHHHHHHCC

alpha-G

Muscle alignment of 4 sequences
