# Supplementary material for: A Novel Protein Kinase-Like Domain in a Selenoprotein, Widespread in the Tree of Life
Source: PLoS One. 2012 Feb 16;7(2):e32138. doi: 10.1371/journal.pone.0032138 (PMC3281104; doi:10.1371/journal.pone.0032138)
Supplement: Figure S3 — Multiple sequence alignment (MUSCLE) of selected eukaryotic SELO proteins (used for construction of the tree shown in Fig. 2 ). Identifiers: NCBI gi numbers. (DOC) [file pone.0032138.s003.doc]

10 20 30 40 50 60 70 80 90 100 110 120 130 140 150

....|....| ....|....| ....|....| ....|....| ....|....| ....|....| ....|....| ....|....| ....|....| ....|....| ....|....| ....|....| ....|....| ....|....| ....|....|

*gi_308808097_[Ostreococcus_* E-V-QVKGGG KTTRTRDGGD GRCTLRSTIR EFLCAEILAA LDVPTTRLMC VISTNE--GV VRR--TKANS ARL--EYGGM SVRVCEAPLV RFGTFELPAS RRDF-DC--V RALADHVIAS DLFVGE---- ----IDDVDR ---HGALLCA

*gi|298715152_[Ectocarpus_si* E-I-QLKGIG QTPFSR-GGD GKAVLGACLR EFLFSEAFLG LGLPAAVAVS VCSSGE--SA TIRDSDPKNG GTFKRARGAV LCRTAPS-FL RFGSFELPAR RGDV-TL--V RKLADYCL-R HLSPHF---E SSLLMGARND ---YVELLVA

*gi|323452602_[Aureococcus_a* E-A-QLKGSG PTPFRR-RGD GFAVVRSSTR EFLASEAMAA LGVPTTRALA LCASAS--DV AER---PWYS DRTAVERRAI TTRVARS-FV RVGSLELFAR RGEPRARREL EALFRYAYDR EGLGPA---G AALE----DA ---AVAAATE

*gi_224011106_[Thalassiosira* E-L-QLKGAG QTPFCR-GAD GRAVLRSSIR EFLASEAMHH LGVCTTRALS LVVSDGANGD TSN--RPWYS DNAKNQRCAI TTRVASS-FV RIGHLDLFAR RAKN-DK--L EDMMWHACYR EYYNDA---Y EPYWKDAKSA ---AMALMNG

*gi_219126041_[Phaeodactylum* E-L-QLKGAG TTPFSR-SGD GRAVLRSSVR EFLASEAMYA LGVDTTRALS LVISDS--ET ISR---PWYN QASSDEKTAI TCRVSMS-FI RIGHFDLYAR RTTA-EWHEL EQLIWHTC-F REFRTECYDP FYPRRNIAAA ---AALLLDL

*gi_219124584_[Phaeodactylum* E-L-QLKGGG TTPFHR-GAD GRAVLRSSIR EFLASEAMHS LNVSTTRALS LVVSEL--DT ISR---PWYS DNSAQEKTAI TCRVATS-FL RVGHLDLFAR RTSTLEWKEL EDLVWHAC-Y REYRHA---A YEPFRDIAAA ---AHVLLKQ

*gi_145355852_[Ostreococcus_* E-L-QLKGGG RTPFCR-GAD GRAVLRSSIR EFLASEAMHA LGVDTTRALC LIESVR--GT TAR--RPWYS PTSDEEPCAI TTRVAPS-FM RIGHIDLFSR RKEQ-----L KKIIRHAA-F REFPET---I EEHGEDMAKV ---TRSMLEK

*gi_308813832_[Ostreococcus_* E-L-QLKGGG RTPFCR-GAD GRAVLRSSIR EFLASEAMHA LGVDTTRALC LIESER--GS TAR--RPWYS SRSDEEPCAI TTRVAPS-FM RIGHIDLFSR RKEQ-----L EKIVRHAA-F REFPET---I EQHGDDTAAM ---TRAMLEK

*gi_255074165_[Micromonas_sp* E-M-QLKGGG PTPFCR-GAD GRAVLRSSIR EFLASEAMHA LGVDTTRALS LVVSRG--GD VAR--RPWYD PNADKETCAI TTRVAPS-FT RVGHVDLFGR RGTA-QMDEL RKMVRHAI-F REFPEI---L NESSDVTPEQ ---VAAFLVS

*gi|323455901_[Aureococcus_a* E-L-QLKGGG QTPFCR-GAD GRAVLRSSLR EFLASEAMHF LGVETTRALS LVVS-G--GE TTR--RPWYS GRQADETCAI TCRVAPS-FV RVGHVDHFAR RKRE-----H KLMVQHAI-A REYPDLLLEH RDETDELRTA ---ALAFAAR

*gi_303290328_[Micromonas_pu* E-M-QLKGGG TTPFCR-GGD GRAVLRSSVR EFLASEAMHA LGVSTTRALS LVVSDG--GD TVR--RPWYN PKSDK----- ---------- ---------- ---------- ---------- ---------- ---------- ----------

*gi|321463811_[Daphnia_pulex* E-L-QLKGAG KTPYSR-NGD GRAVLRSSIR EYLCSEAMHA LGIPTSRAAA IVVS-K--DM VVR--DQFYN GRMKYEPTAV VLRLAPT-WF RIGSLEILTR EKEI-KN--L KQVVDFTI-E HHMPTI---- ------PQGN ---YLKFLET

*gi_196009079_[Trichoplax_ad* A-L-QLKGSG KTPYSR-DGD GRAVLRSSIR EYLVSEAMYH LGIPTTRAAS IVTS-D--EP IWR--DQFYD GHPRAEKAGI VLRLAPS-WF RFGSIEILHY NQEF-HL--L NRLVDVII-N LHYPHL---- ----SDDNRK ---YIKFYAE

*gi_156359336_[Nematostella_* E-L-QLKGSG KTPYSR-HGD GRAVFRSSVR EFLASEAMHY LGVPTSRVAS LVVS-D--EQ VWR--DQFYD GHPIREKAAV VLRLAKS-WF RIGSLEILTN NGET-DL--L RKVVDFVI-E QHFNKI---- ----KDSKEK ---YLEFFSQ

*gi_115638715_[Strongylocent* E-L-QLKGSG KTPYSR-RGD GRAVLRSSVR EFLGSEAMFY LGVSTSRALS LVVS-E--DP IWR--DQFYD GNPRQEKAAV VLRLAPS-WF RIGSLEILAK NREI-DL--L RSLVDFII-K HHFKEI---D SEDD----DR ---YLAFYSE

*gi_291227954_[Saccoglossus_* E-L-QLKGSG LTPYSR-RGD GRAVLRSSIR EFLCSEAMYH LGIPTSRALS VIVS-G--DP VWR--DQFYD GHAKTEKAAV VLRLAKS-WF RIGSLEILAM KREI-KL--L RRLTDFVI-E NYFPSI---D ISDE----NK ---YLSLFSE

*gi_260794897[Branchiostoma_* E-L-QLKGSG LTPYSR-RGD GRAVLRSSVR EFLCSEAMYH LGIPTSRAAT LIVS-D--DP VIR--DQFYN GHPKKERGAV VLRLAKS-WF RIGSLEILAA NQET-QL--L KQLVDFTI-Q QYFTDI---Y ETLS----DR ---YLTFFSD

*gi_319738607_[Xenopus_(Silu* E-L-QLKGSG RTPYSR-GGD GRAVLRSSVR EFLCSEAMHY LGIPTSRA-- ---------- ---------- ---------- ---------- ---------- ---------A RKLVDFII-T NYFPSI---N SKAP----NR ---TLTFFSI

*gi_194227089_[Equus_caballu* ----HLIGI- ---YMNSHGD GRAVLRSSVR EFLGSEAVHH LGIPTSRAAS LVVS-D--DE VWR--DQFYD GNVVKERAAV VLRVAKS-WF RIGSLEILAH YGEL-DL--L RTLLDFII-Q EHFPSV---D VGEP----NR ---YVDFFSV

*SELO2_BOS_gi_297481447_[Bos* E-L-QLKGSG KTPYSR-NGD GRAILRSSLR EFLCSEAMHY LGIPTSRAAS LVVS-D--DV VWR--DQFYN GNLTKERGAV VLRVAKS-WF RIGSLEILTH SGEL-DL--L RMLLDFII-Q EYFPLV---D VKEP----NR ---YVDFFSI

*gi_327274681_[Anolis_caroli* E-L-QLKGSG RTPYSR-NGD GRAVLHSSVR EFLGSEAVHY LGIPTSRAAS LVVS-D--DD VWR--DRLYN GNVKKER--- ---------- ---------- ---------- ---------- ---------- ---------- ----------

*SELO2_CHICK_gi_118085664_[G* E-L-QLKGSG KTPYSR-NGD GRAVLRSSVR EFLCSEAMHY LGIPTSRAAS LVVS-D--DA VWR--DQFYN GNIKKERGAI VLRLAKS-WF RIGSLEILAH SGEL-DL--L RRLLDFII-H EHFPSI---V PNSS----DR ---YLEFFST

*YDIU_ECOLI_gi_3183285_[Esch* ----HLKGAG LTPYSR-MGD GRAVLRSTIR ESLASEAMHY LGIPTTRALS IVTS-D--SP VYR--ETA-- -----EPGAM LMRVAPS-HL RFGHFEHFYY RRES-EK--V RQLADFAI-R HYWSHL---- ----ADDEDK ---YRLWFSD

*gi_159480380_[Chlamydomonas* K-LGKRKGKG PTHGVR-RAD GRAVLRSSLR EFVASEAMAA LGVPTTRALS LVGT-G--DK VLR--DMFYN GNAKMEQGAV VCRVAPS-FV RFGTFQLPVS RGAG-EVGLV KMAADWVI-K HHMPHL---A GEGENKSPEP ---YLGLLRE

*gi_302841364_[Volvox_carter* E-L-QLKGAG KTPYSR-RAD GRAVLRSSLR EFVCSEAMAA LGVPTTRALS LVGT-G---- ---------- -----GPGAV VCRVAPS-FM RFGTFQLPVS RGLG-EVGLV KMAADWVI-K YHNPHL---A SDLSSDSPQP ---YLDLLRE

*gi_115467830_[Oryza_sativa_* E-L-QLKGCG KTPYSR-FAD GLAVLRSSIR EFLCSEAMHG LGIPTTRALC LVET-G--KS VVR--DMFYD GNSKEEPGAI VCRVAPS-FL RFGSYQIHAT RDKE-DLEIV RHLADYTI-R HHYPHL---E NIKKDLTSNK ---YAAWAVE

*gi_293335415_[Zea_mays]*  E-L-QLKGCG KTPYSR-FAD GLAVLRSSIR EFLCSEAMHG LGIPTTRALC LVET-G--KS VVR--DMFYD GNAKEEPGAI VCRVAPS-FL RFGSYQIHAS RGKE-DIEIV RRLADYTI-H HHFPHL---E NMKKDLTSNK ---YAAWAVE

*gi_30684227_[Arabidopsis_th* E-L-QLKGAG RTPYSR-FAD GLAVLRSSIR EFLCSETMHC LGIPTTRALC LLTT-G--QN VTR--DMFYD GNPKEEPGAI VCRVSQS-FL RFGSYQIHAS RGKE-DLDIV RKLADYAI-K HHFPHI---E SMDRDLTSNK ---YAAWIVE

*gi_224053020_[Populus_trich* E-L-QLKGSG RTPYSR-FAD GLAVLRSSIR EFLCSEAMHC LGIPTTRALS LVTT-G--KY VTR--DMFYD GNAKEEPGAI VCRVAPS-FL RFGSYQIHAS RGKE-DLEIV RALADYAI-R HHFPHI---E NMNKDLTSNK ---YAAWTVE

*gi_255544744_[Ricinus_commu* E-L-QLKGAG KTPYSR-FAD GLAVLRSSIR EFLCSEAMHH LGIPTTRALC LVTT-G--KY VTR--DMFYD GNPKEEPGAI VCRVAQS-FL RFGSFQIHAS RGKE-DFGIV RALADYAI-R HHFPHI---D NMTKDLTSNK ---YAAWTVE

*gi_225435594_[Vitis_vinifer* E-L-QLKGAG RTPYSR-FAD GLAVLRSSIR EFLCSEAMHS LGIPTTRALC LVTT-G--KY VTR--DMFYD GNPKEEPGAI VCRVAQS-FL RFGSYQIHAA RGKE-DLGIV RALADYTI-R HHFPHI---E NMTRDLTSNK ---YAAWSVE

*gi_168047679_[Physcomitrell* E-L-QLKGAG KTPYSR-TAD GLAVLRSSVR EYLCSEAMYH LGVPTTRALS LVTT-G--EG VLR--DMFYD GNVKMEPGAV VCRVSPS-FI RFGSFQIHAA RDKA-DLPIV KQLADYTI-H HHYPDF---E DLPFDTSKNK ---YSAWFTE

*gi_302804871_[Selaginella_m* E-L-QLKGAG KTPYSR-MAD GLAVLRSSVR EFLCSEAMHH LGIPTTRALC LVTT-G--DD VLR--DMFYD GNAKMEPGAV VCRVAPS-FL RFGSYQIHAA REDS-KL--V RLLADYTL-K YHFPDLPDEE ELEIQISKNK ---YAAWFVK

*gi_254564783_[Pichia_pastor* E-I-QLKGSG KTPFSR-FAD GKAVVRSSVR EYVISESLHA LGIPTTRALA IVTLPH--TW AQR--YAA-- -----ENCAI TTRFAPS-WI RIGTFDLYRW RGDR-KG--M RTLSDFVI-S DVFGGK---E AVLNFGEFSI ---YDLMYLE

*gi_68484234_[Candida_albica* E-I-QLKGAG KTPFSR-FAD GKAVLRSSIR EYIISEHLHA IGVPTTRALA LTYLPS--TL AQR--HGA-- -----ERCAI VARFAES-WV RMGTFDLYRW RGDR-EG--I RDLSDYVI-N ELFTIN---G VKFQLGELTD ---YDKMYYE

*gi_154318896_[Botryotinia_f* E-L-QLKGAG ITPYSR-FAD GKAVLRSSIR EFIVSEALNG LKIPTTRALS LTLLPF--SK VRR--EIT-- -----EPGAI VARFAES-WL RIGTFDILRA RGDR-AL--I RELCTYIA-E NVFQGW---E SLPGGLEENR ---FTRLYRE

*gi_255931617_[Penicillium_c* E-L-QLKGAG RTPYSR-FAD GKAVLRSSIR EYVVSEALSA LGIPTTRALS LTLIPN--AK VLR--ERL-- -----EPGAI VARFAES-WL RIGTFDLLRV RGDR-EL--I RKLATYVA-E DVFNGW---E SLPADVQENR ---FARLYRE

*gi_317029685_[Aspergillus_n* E-L-QLKGAG RTPYSR-FAD GKAVLRSSIR EYIVSEALSA LGVPTTRALS ITLLPQ--SK VLR--ERI-- -----EPGAI VARFAES-WL RIGTFDLLRA RGDR-EL--I RHLATYIA-E EVFQGW---E ALPAGSEENR ---FARLYRE

*gi_327297586_[Trichophyton_* E-I-QLKGAG LTPYSR-FAD GKAVLRSSIR EFIVSEALNA LGIPTTRALS LTLLPN--CS VRR--ERL-- -----EPGAI VTRFAES-WI RIGTFDLLRA RSDL-KL--T RQLATYVA-E DVFHGW---E SLPAGAEENR ---FARLYRE

*gi_302510829_[Arthroderma_b* E-I-QLKGAG LTPYSR-FAD GKAVLRSSIR EFIVSE---- ---------- ---------- ---------- ---------- VTRFAES-WI RIGTFDLLRA RSDL-KL--T RRLATYVA-E DVFPGW---E SLPAGAEENR ---FARLYRE

*gi_261192888_[Ajellomyces_d* E-L-QIKGAG RTPYSR-FAD GKAVLRSSIR EYVVSEALNA LGIPTTRALS LVLLPN--SK VRR--ERL-- -----EPGAI VTRFAQS-WI RIGTFDLPRS RGDR-DL--T RKLATYVA-E DVFPGW---E SLPAGAEENR ---FTRLYRE

*gi_119196335_[Coccidioides_* E-I-QLKGAG RTPYSR-FAD GKAVLRSSIR EYVISEALNA LGIPTTRALA LTLLPD--VA VRR--EKI-- -----EPGAI VTRFAES-WL RIGTFDLLRA RGDR-DL--T RKLANYIA-E DVFSGW---E SLPAGAEQNR ---FSRLYRE

*gi_164428165_[Neurospora_cr* E-V-QLKGAG MTPYSR-FAD GKAVLRSSIR EFIVSENLHA LGIPSTRALA ISLLPH--SR VRR--ETM-- -----EPGAI VVRMAQS-WL RFGNFDILRA RGDR-KL--V RQLATYIG-E EVFGGW---D KLPGGAEENR ---FHRLYRE

*gi_145608380_[Magnaporthe_o* E-V-QLKGAG LTPYSR-FAD GKAVLRSSIR EFVASESLHA LGVPTTRALA LSLLPH--QK VRR--ETV-- -----EPGAI VVRFAQS-WI RLGTFDLLRA RGDR-DL--I RKLATYVA-E DVLGGW---E NLPGEMAENR ---FVRLYRE

*gi_19115652_[Schizosaccharo* E-I-QVKGAG RTPYSR-FAD GKAVLRSSIR EYLCCEALYA LGIPTTQALA ISNLEG--VV AQR--ETV-- -----EPCAV VCRMAPS-WI RIGTFDLQGI NNQI-ES--L RKLADYCL-N FVLKDG---- -FHGGDTGNR ---YEKLLRD

*FMP40_YEAST_gi_6325034_[Sac* QTF-QLKGAG MTPFSR-FAD GKAVLRSSIR EFIMSEALHS IGIPSTRAMQ LTLLPG--TK AQR--RNQ-- -----EPCAV VCRFAPS-WI RLGNFNLFRW RHDL-KG--L IQLSDYCI-E ELFAGG---T QFEGISTLSK ---YDEFFRH

*gi_50303343_[Kluyveromyces_* --V-QLKGSG LTPYSR-FAD GKAVVRSSIR EFLICESLHA IGIPSSRALQ ISSLPK--TR ARR--SVF-- -----EPCAV ITRFAPS-WI RIGNFDLFRW KQDD-EG--L LKLADFSI-N HVFEGL---K DYRDDDGLTN ---YDKLFRH

*gi_302308501_[Ashbya_gossyp* QTL-QLKGSG MTPFSR-FAD GKAVLRSSVR EFIVSEALAA IGIPSTRALQ LTLLPK--TR ARR--TMF-- -----EPCAV VARFAPS-WI RLGNFDMFRW RGDD-HG--L LQLVDYCI-Q HVFAAG---R EFPTVAGVTT ---YDLFFRH

*gi_290979991_[Naegleria_gru* E-L-QFKGAG HTPFSR-HAD GRAVLRSSIR EFLGSEFMDS LGIATTRAFS LVRSKE--KA VLR--DEFYD NNPKYEYGAI VLRVAPT-FV RFGSFDIFNY REEK-KN--I EVLARYVI-K NHFPHL---W IN--GDLTEL ---KEKFSKE

*gi_229593872_[Tetrahymena_t* E-L-QLKGSG ITPYSR-FAD GNAVLRSSIR EYLCSEAMHF LNIPTTRAAS ITIT-E--DQ AMR--DPLYN QQIVYEKCAV VLRLSPT-FI RFGSFQICNK QGE--QM--I PELLDFII-K NHYPEF---- ----NGKEDK ---YMLFLQE

*gi|145516136_[Paramecium_te* E-L-QLKGSG LTPYSR-FAD GKAVIRSSVR EYLCSEFMFH LNIPTTRAAS LVIT-D--SK AER--DIFYD GHPILENCAV VLRIAQT-FL RFGSFEVEID LNPKNTI--I PQLWDYCK-K QYFGDK---- ---------- ----ENPFQE

*gi_294872672_[Perkinsus_mar* E-I-QLKGSG KTPFSR-SAD GRKVLRSTIR EFLCSEHMHA LGIPTTRAAA VSVSFE--DQ VIR--DINYD GNAKLEPTAV VVRLAET-FL RFGSFEIFKS TGDT-AL--L QKLVDFVI-N NYYEAE---C ADIEETSVKK ---CEQFFQA

*gi_115916063_[Strongylocent* ----NFAGQ- -------LGD GAAMLVC--- --ILFESYFR SGLGNN---- --------HD ANK------- -----E---- ---------- QFGSFEIFKP TGRK-DI--L EKMLDYSI-Q SFYPKI---Y EDHCEDLVQR ---NLAFYRE

*gi_167537910_[Monosiga_brev* E-L-QFKGAG LTPFSR-QAD GRKVLRSSIR EFLCSEAMHA LNIPTTRAGS LITS-D--TR VVR--DIFYT GSLIQERATV ITRLAPS-FL RFGSFEVVKE KGQV-EL--T KKLLDYLL-A HHFADI---W SQDS-SPEDK ---FAEFLAE

*gi_256073786_[Schistosoma_m* E-L-QLKGAG LTPFSR-QGD GRKVLRSSLR EFLCSEAMYY LGIPTTRAAS IITS-D--TL VER--DMFYT GDSITEKASI TSRVAKT-FI RFGSFEISKS PGNL-TI--L SQLTNYVI-Q QFYPHI---W SDYSNDIMNC ---YLEFFKE

*gi_159483357_[Chlamydomonas* E-L-QFKGAG KTPYSR-QAD GRKVLRSSLR EFLCSEAMYN LGIPTTRAGT CVTS-D--SK VVR--DIKYD GNAILERATT ITRIAPT-FL RFGSFEIFKP TGHEAAI--L PVMLHHAI-R TYYPAI---W AAHDGDRIAM ---YLDWIKE

*gi_302845399_[Volvox_carter* E-L-QFKGAG KTPYSR-QAD GRKVLRSSLR EFLCSEAMYH LGVPTTRAGT CVTS-D--TR VVR--DVFYD GNAILEKATI ITRIAPT-FL RFGSFEIFKP VGQEVAM--L PTLLHHTI-R TYFPDI---W ASHQGLEVAM ---YLDWLIE

*gi_298286503_[Ciona_intesti* E-I-QLKGAG QTPYSR-SAD GRKVLRSTIR EFLCSEAIFH LGIPTTRAGT VVVS-D--DK VVR--DMFYD GKAKLENCAV VLRLAPS-FL RFGSFEIFKP IGMT-GI--L PTMLQYAL-D NFFKEV---D QALP--KVEQ ---YLAMYKE

*gi_221116553_[Hydra_magnipa* E-L-QLKGAG LTPYSR-NAD GRKVLRSSIR EFLCSEAMFY LGVPTTRAGS CITS-D--TR VVR--DIFYD GNPIMERCTI VSRIAPS-FI RFGSFEIFKP LGKD-DI--L HTLLEYVV-S TFYPEI---W QTHSGNKEKA ---YLDFFKE

*gi_340370931_[Amphimedon_qu* E-L-QLKGSG KTPYSR-HAD GRKVLRSSIR EFLCSEAMHY LGIPTTRAGS CITS-E--SL VAR--DIFYN GNVIQEQATV ISRIAPT-FI RFGSFEIFKT RGRD-DI--F HLLLDYVT-E HFYPEI---Y KSHLDDIEAR ---TAGFFNE

*gi_156406460_[Nematostella_* E-M-QLKGSG LTPYSR-QAD GRKVLRSSIR EFLCSEAMYH LGIPTTRAGS CVTS-D--TK VIR--DIFYN GNAKSEKATI ILRIAPT-FI RFGSFEIFKP IGRK-DI--L LQLLEYTI-K TFYPKI---Y DLHS-SPEER ---YLAFYKD

*gi_195999240_[Trichoplax_ad* E-I-QFKGSG LTPYSR-HAD GRKVLRSSIR EFLCSEAMHH LGIPTTRAGS CITS-D--SE VLR--DIYYS GNPIKEKATV ILRIAPT-FL RFGSFEIFKP LGRK-DI--L IQLLEYTI-N THFPHV---A AKYPDSDKER ---YLAFFEE

*gi_260794380_[Branchiostoma* E-I-QLKGAG LTPYSR-TAD GRKVLRSSIR EFLCSEAMHH LGIPTTRAGS CVTS-D--SK VLR--DVYYN GNASYERCTI VLRIAQT-FL RFGSFEIFKP TGRN-DI--L ITMLDYAI-K TFFPEI---Q EAHA-DSEER ---YLAFFRE

*gi_319738592_[Xenopus_(Silu* E-I-QLKGAG LTPYSR-QAD GRKVLRSSIR EFLCSEAMSH LGIPSTRAGS CVTA-D--ST VIR--DIYYD GNPKKEKCTV VSRIAPT-FL RFGSFEIFKP TDRN-DI--R IQMLDYVI-R TFYPDI---Q EKHAGNNTEK ---NAAFFRE

*gi_327273185_[Anolis_caroli* E-A-QLRGAG LTPFSR-QAD GRKVLRSSIR EFLCSEAMFH LGIPTTRAGT CVTS-D--SE VIR--DIFYD GNPKKEKCTV VLRIAPT-FI RFGSFEIFKP ANRN-DI--R IQMLDYVI-S TFYPEI---L EAHSDNKVER ---NTAFFRE

*SELO_CHICK_gi_169234793_[Ga* E-L-QLKGAG ITPFSR-QAD GRKVLRSSIR EFLCSEAMFH LGIPTTRAGT CVTS-D--SE VVR--DIFYD GNPKKERCTV VLRIAST-FI RFGSFEIFKP PNRN-DI--R IQMLDYVI-G TFYPEI---Q EAHADNSIQR ---NAAFFKE

*gi_334347697_[Monodelphis_d* E-L-QLKGAG LTPFSR-QAD GRKVLRSSIR EFLCSEAMFH LGIPTTRAGS CVTS-E--SK VIR--DIYYD GNPKYESCAV VLRIAST-FL RFGSFEIFKP PGRN-DI--R VQMLDYVI-G SFYPEI---Q AAHARDSMQR ---NLAFFRE

*SELO_BOS_gi_319803072_[Bos_* E-L-QLKGAG PTAFSR-QAD GRKVLRSSIR EFLCSEAMFH LGVPTTRAGS CVSS-Q--ST VVR--DAFYD GNPRPEPCAV VLRLAPT-FL RFGSFEIFKP RGRD-DI--R LQMLDYVI-S TFYPEI---Q ACHPGDHVQR ---HAAFFRE

*gi|81295807_[Mus_musculus]*  E-L-QLKGAG PTPFSR-QAD GRKVLRSSIR EFLCSEAMFH LGIPTTRAGA CVTS-E--ST VMR--DVFYD GNPKYEKCTV VLRIAPT-FI RFGSFEIFKP PGRD-DI--R VQLLDYVI-S SFYPEI---Q AAHTTDNIQR ---NAAFFRE

*SELO_HUMAN_gi_32880229_[Hom* E-L-QLKGAG PTPFSR-QAD GRKVLRSSIR EFLCSEAMFH LGVPTTRAGA CVTS-E--ST VVR--DVFYD GNPKYEQCTV VLRVAST-FI RFGSFEIFKS AGRN-DI--R VQLLDYVI-S SFYPEI---Q AAHASDSVQR ---NAAFFRE

*gi_47600579_[Escherichia_co* N-Y-QVKGIG SNPLER-HSN GALGAVHAIY EALWGEVLAQ LPYSAVRVRA VLLT-D--LY TEK--A--FE RSGRKSRRAL LVR--EP-VV RPAHFERAPY FHDA-CR--V RSVIHKLL-P VPPEEI---D AEARTDPRIY ---CIEGLCE

*gi_338721443_[Equus_caballu* E-L-QLKGAG PTPFSR-QAD GRKVLRSSIR EFLCSEAMFH LGIPTTRAGA CVTS-Q--ST VVR--DAFYD GNPKYEKCTV VLRIAST-FL RFGSFEIFKS TGRN-DI--R VQMLDYVI-G SFYPEI---Q AAHASDSVQR ---NAAFFRE

*gi_71003666_[Ustilago_maydi* E-I-QLKGAG RTPYSR-FAD GLATLTSSVR EYLCSEAMGA LGIPTSRALS IVSLPE--LH VVR--ERL-- -----NIAAI TTRLCPS-WL RIGSFQIHSS RNEW-ES--V RVLGEYVS-H DLFGFEDVVK GGHVGDGAQR PVWARRMVEE

*gi_58271330_[Cryptococcus_n* E-I-QLKGAG RTPFSR-ADD GLAHLRSGVR EFLGCEAVAA LGIPTTRALT LLTIPLPYLP VFR------E GTI--HPSSL LTRVAPS-FI RVGHFEALNP PDQTDEEGED EGNGNFEG-L RDLGEW---M KEI-MEVKEG ---WKSWVDE

160 170 180 190 200 210 220 230 240 250 260 270 280 290 300

....|....| ....|....| ....|....| ....|....| ....|....| ....|....| ....|....| ....|....| ....|....| ....|....| ....|....| ....|....| ....|....| ....|....| ....|....|

*gi_308808097_[Ostreococcus_* AARRNAITVA RWMSIGFVHG VMNTDNTTVC GTTID--LGR FGFME-AYDE EFCSNPDD-E TK-MYAFGRQ RSVARWNVER LCDAF--ERV VPASA---HR SALCAFDDAF ENE-TKRLNE RKFGVR---- --DD----DG VVS---SAFH

*gi|298715152_[Ectocarpus_si* IVQATARMVA GWQAMGFCHG VLNTDNFTLL GLGLD--FGP CSFME-AYDP TWSPNEGD-S AL-RYAFRNQ PDVSAWNCER LAEAF--SPI VGVSG---VA EANAAFSPAF DAA-YTVHLQ KKLGLSAG-- EMQEKLPPDA QFV---LSFF

*gi|323452602_[Aureococcus_a* TRGRFASLAC HWLRVGYAQS NYNSDNSLVG GATVD--YGP FGFVE-RYDK TWAMWV---G SGDHFAFGNA LEAARRNWAT LVDAL--RP- ---------- ---------- ---------- ---------- ---------- ----------

*gi_224011106_[Thalassiosira* TMNTLSRMVA NWVRVGFVQG NFNADNCLVG GRTMD--YGP FGFLD-VYHP LSAKWT---G SGEHFGFMNQ PKAGYANFAV LVESL--LPI IEANGDKVRD EMLKKAQTVF SEA-VDNAMS SKMGIQ-V-- GPPEMAKEAD ELW---EEIE

*gi_219126041_[Phaeodactylum* AAEKIATMVA GWIRVGFAQG NFNADNCLVA GRTVD--YGP FGFVE-EFDP TFSKWT---G SGTHFGFMNQ PSAALANYKI LVESV--VPV IAAQTERIRT SFLERAQILF EKA-VSEVFR IKLGFS-R-- DQKE----GD RLW---NSLQ

*gi_219124584_[Phaeodactylum* AADRIAAMVA DWVRVGFIQG NFNADNCLVG GRTMD--YGP FGFLE-YYHP TAAKWT---G SGEHFGFLNQ PSAGFANFKV LAESV--VPV IAAAHSKILK NVLDDAAKVF QNK-VDETFR KKLGFT-V-- EQDI----GD DVW---EALE

*gi_145355852_[Ostreococcus_* SGKKIAKMVA GWIRVGFCQG NFNADNCLVG GRTMD--YGP FGFMD-KYDP SFAKWT---G SGDHFAFMAQ PKAGLTNFAV LAVSC--APL LAGGS-DEAT ELVREMEATF ENE-LNDVFR AKLGFA---- PNEDSVRVAR DLFRSENGLE

*gi_308813832_[Ostreococcus_* SGAKIAKMVA GWLRVGFAQG NFNADNCLVG GRTMD--YGP FGFMD-RYDP GFAKWT---G SGEHFAFMAQ PDAGITNFAV LAVSC--APL LKGGS-KEAQ DIVRDAQPVF ARA-VDDTFR TKLGFA-SES SMDV----AR ELFRAEDGLE

*gi_255074165_[Micromonas_sp* SGAAIADLVC GWLRVGFCQG NFNADNCLVG GRTMD--YGP FGWMD-AYDP LFAKWT---G SGEHFAFMNQ PGAGLANFAV LAASC--APL LKGGE-DEAQ AIVERMQGEM EQK-VADVWR VKLGLR---- --ESQRAAAA KLF---DKLE

*gi|323455901_[Aureococcus_a* ARDALALLAA GWLRVGFCQG NFNADNCLVG GRTMD--YGP FGFMD-KYDP SFAKWV---G SGDHFAFAAQ PGAALANFRT LAKAL--LP- ---------- ---------- ---------- ---------- ---------- ----------

*gi_303290328_[Micromonas_pu* --KGGATMVA GWLRVGFCQG NFNADNCLVG GRTMD--YGP FGFVDPKYDP LFAKWT---G SGEHFAFANQ PAAGLANFAV LASSV--APL LRDGD-EEAN ALVAEARLYF DEA-VSDVRR AKLGFS---- -EDY----DA ETA---STLE

*gi|321463811_[Daphnia_pulex* VLEQSAALVS LWMAHGFTHG VLNTDNMSLL SITID--YGP FGFLD-SYNP SFVPNHSD-D EG-RYSYLNQ PKIFKWNMAR LADAL--QPL LSAEEQKEAA ATIGRFDEIY QQQ-FISIFR RKLGLS-K-- AAKD----ED KLV---QLLL

*gi_196009079_[Trichoplax_ad* IINTTASLIA QWQSVGFTHG VCNTDNFSIL SLTID--YGP FGFLD-EYND DFISNTSD-D DG-RYRFRFQ PNVAYFNLDK LRIAL--SSL ISEVD---GQ KELSNYKRIY RRH-YLHIMR KKLGLK-G-- SNKK----DT KLI---TQML

*gi_156359336_[Nematostella_* VVTKTAHMIA IWQALGFAHG VCNTDNFSLL SMTID--YGP FGFMD-TYNS DFVPNTSD-D EG-RYSFSNQ PSAGQYNLAK LLDAL--SPI IDLARYLAGK KILQRYAAEF NNC-FMDLHR QKLGLV-G-- RRDE----DD MLI---KSFL

*gi_115638715_[Strongylocent* IINKTAYMIA KWQSIGFAHG VMNTDNFSLL SITID--YGP FGFLD-AYDP QYIPNTSD-D EG-RYSYEKQ PEVGLFNMRK LADAL--APL LTLPQRKQLP IITSGYVDIY KAR-FMELFR KKLGLI-G-- AEEQ----DE YIV---AMLL

*gi_291227954_[Saccoglossus_* IVSQTADLMA RWMSVGFAHG VMNTDNFSLL SITID--YGP FGFLD-DYNP SFIPNTSD-D EG-MYSYENQ PDIGHFNMNR LRAAL--WPL WNNKQKQLSE MILQGYIDIY KTR-FMEIFR GKLGFL-S-- TDDK----DE YII---GLLL

*gi_260794897[Branchiostoma_* VVSQTAEMIA LWQSVGFAHG VCNTDNFSLL SITID--YGP FGFMD-SYDP EFVPNTSD-D TG-MYSYENQ PDVGLFNLDK LREAL--ASL LTEQQRFQMT KILELYPDIY KTK-YMEILR RKMGML-G-- EEED----DA MIA---AVLF

*gi_319738607_[Xenopus_(Silu* VVSETANLIA SWMSVGFAHG VCNTDNFSLL SITID--YGP FGFME-SYDA DYVPNTSD-D EG-RYSIGNQ ANVAMFNLNK LRLAL--NPL LDSKQQQQAS QVLRGFPDLY YKR-FTELFR AKLGIL-G-- ENDQ----DL ALI---SSFL

*gi_194227089_[Equus_caballu* VVSETAQLIA LWTSVGFAHG VCNTDNFSLL SITID--YGP FGFME-AYNP DFVPNTSD-D ER-RYKIGNQ ANIGMFNLNK LLQAL--NPL LDPRQKQLAA LILEGYPDLY YTR-FRELFK AKLGLL-G-- ERKG----DE DLI---AFLL

*SELO2_BOS_gi_297481447_[Bos* VVFETAQLIA LWMSVGFAHG VCNTDNFSLL SITID--YGP FGFME-AYNP DFVPNTSD-D ER-RYKIGNQ ANIGMFNLNK LLQAL--NPL LNPRQKQLVT QILKEYPVLY YTR-FRELFK AKLGLL-G-- KSEG----DD DLI---AFLL

*gi_327274681_[Anolis_caroli* ---------- ---------G VCNTDNFSLL SVTID--YGP FGFMD-SYDP DFVPNTSD-D ER-RYKIGNQ ANVGLFNLNK LLQAL--NRL LDSRQKQLAS QILMGYSEQY YRC-FMEIFK RKLGLL-G-- DDDG----DN YLI---AFLL

*SELO2_CHICK_gi_118085664_[G* VVSETANLIA LWMSVGFAHG VCNTDNFSLL SITID--YGP FGFMD-SYDP NFVPNTSD-D EG-RYKIGNQ ANVGLFNLNK LLQAL--KPL LDPRQKQLAS HILEEYGQTY YVR-FTELFK RKLGLL-G-- DSED----DN YLI---AFLL

*YDIU_ECOLI_gi_3183285_[Esch* VVARTASLIA QWQTVGFAHG VMNTDNMSLL GLTLD--YGP FGFLD-DYEP GFICNHSD-H QG-RYSFDNQ PAVALWNLQR LAQTL--SPF VAVDA---LN EALDSYQQVL LTH-YGERMR QKLGFM-T-- EQKE----DN ALL---NELF

*gi_159480380_[Chlamydomonas* ACARTGRLVA QWQALGFVHG VLNTDNMSIL GLTID--YGP YGFLD-VFDP DWTPNLTD-A SGRRYSYRNQ PEAGQFNVVM LGNALLAADL LGREA---AT EALVGYSEVL STT-YNQLMA AKLGLK---- --EY----DR TLA---QELM

*gi_302841364_[Volvox_carter* VTCRTATLVA AWQSLGFVHG VLNTDNMSIL GLTID--YGP FGFLD-KFDP DWTPNLTD-A GGRRYSYRNQ PEAVQFNLVM LGNALLAADL VPREG---AE EVLREYSKVL SES-YNARMA AKLGLR---- --EY----DM TLT---HELM

*gi_115467830_[Oryza_sativa_* VAERTAFLIA RWQGVGFTHG VLNTDNMSVL GLTID--YGP FGFLD-AFDP SYTPNTTD-L PGKRYCFANQ PDVGLWNIAQ FTSPLTAAEL ISKDE---AN YVMERYGTKF MDE-YQSIMT RKLGLP---- --KY----NK QLI---GKLL

*gi_293335415_[Zea_mays]*  VAERTAYLIA RWQGVGFTHG VLNTDNMSVL GLTID--YGP FGFLD-AFDP SYTPNTTD-L PGKRYCFANQ PDVGLWNIAQ FTGPLSSAEL ISQDE---AN YVMERYGTKF MDE-YQSIMT KKLGLT---- --KY----NK QLI---SKLL

*gi_30684227_[Arabidopsis_th* IAERTATLVA RWQGVGFTHG VLNTDNMSIL GQTID--YGP FGFLD-AFDP SYTPNTTD-L PGRRYCFANQ PDIGLWNIAQ FSKTLAVAQL INQKE---AN YAMERYGDKF MDE-YQAIMS KKLGLT---- --KY----NK EVI---SKLL

*gi_224053020_[Populus_trich* IAERTASMIA SWQGVGFTHG VMNTDNMSIL GLTID--YGP FGFLD-AFDP SFTPNTTD-L PGRRYCFANQ PDIGLWNIAQ FTATLSTAKL ISDKE---AD YAMERYGNKF MDE-YQAMMT RKLGLP---- --KY----NK QLI---SKLL

*gi_255544744_[Ricinus_commu* VAERTASLIA SWQGVGFTHG VLNTDNMSIL GLTID--YGP FGFLD-AFDP SYTPNTTD-L PGRRYCFANQ PDIGLWNIAQ FTATLSEAQL INDKE---AN YAMERYGNKF MDE-YQAIMT RKLGLP---- --KY----NK QLI---SKLL

*gi_225435594_[Vitis_vinifer* VAERTASLVA SWQGVGFTHG VLNTDNMSVL GLTID--YGP FGFLD-AFDP SYTPNTTD-L PGRRYCFANQ PDIGLWNIAQ FTSTLMSAEL INDKE---AN YAMERYGTKF MDE-YQAIMT RKLGLP---- --KY----NK QLI---SKLL

*gi_168047679_[Physcomitrell* IAERTALMIA KWQAVGFTHG VMNTDNMSIL GLTID--YGP FGFLD-AFDP KYTPNTTD-L PGRRYGFANQ PDIGLWNVMQ LANTLYTAEL ITADE--AQY VTIQIYADKF MFL-YQQHMS NKIGLK---- --TY----NK DLL---SKLL

*gi_302804871_[Selaginella_m* VAESTSCLVA MWQAVGFTHG VLNTDNMSVL GLTID--YGP FGFLD-AFDP KYTPNTTD-L PGRRYCFANQ PDIGLWNILQ FGNTLMAAGL LTQEE---LQ YGLNRYADTF MVH-YQQNMT KKLGLK---- --EY----NK DLT---SKLL

*gi_254564783_[Pichia_pastor* IVVRNAKTVS QWQAYGFLNG VLNTDNTSVL GLSID--FGP FAIMD-KFDP DFTSNSED-H TL-RYSHRNT PGAVFWSLTR LGEDM--AEL IGAGPTAVIE LAAILFEHIV TKE-YGQLLM KRLGLSSY-- EEAD----EQ LLL---DPLR

*gi_68484234_[Candida_albica* TVVRNAETTA ICQCYGFLNG VLNTDNTSIL GLTID--FGP FSIMD-KYNP NYTPNSED-H EG-RYGYRNV PTAIWWNLTR LGEDL--AEL VGAGNTKIIE IGGEIYQHAF TKK-YVETFF NRLGLSQN-- PEIQ----RT DVI---VPML

*gi_154318896_[Botryotinia_f* IVQRNARTVA AWQAYAFTNG VLNTDNTSIF GLSID--FGP FAFLD-NFDP SYTPNHDD-H ML-RYSYRNQ PTIIWWNLVR LGESF--GEL IGAGAEALIT RVGEEYKATF LAE-YKRLMT ARLGLKVF-- KESD----FG TLY---SELL

*gi_255931617_[Penicillium_c* IARRNAKTVA AWQAYGFMNG VLNTDNTSIY GLSLD--YGP FAFMD-NFDP QYTPNHDD-H ML-RYAYRNQ PSIIWWNLVR LGESL--GEL IGAGNEKIIE RVGEEFKAVF LNE-YKRLMG QRLGLKTQ-- AESD----FQ NLF---SEML

*gi_317029685_[Aspergillus_n* IARRNAKTVA AWQAYGFMNG VLNTDNTSIY GLSLD--YGP FAFMD-NFDP QYTPNHDD-H LL-RYCYKNQ PTIIWWNLVR LGESL--GEL IGAGEEKVID RTGKEFRMVF LNE-YKRLMS NRLGLRSQ-- KETD----FQ VLF---SELL

*gi_327297586_[Trichophyton_* IVRRNAKTVA AWQAYGFMNG VLNTDNTSIF GLSLD--FGP FASMD-NFDP SYTPNHDD-E ML-RYSYKNQ PSVIWWNLVR LGESF--AQL IGIGDEKLID QAGDEFKTVF LNE-YKRLMS TRLGLKTQ-- KESD----FN ELF---SNLL

*gi_302510829_[Arthroderma_b* IVRRNAKTVA AWQAYGFMNG VLNTDNTSIF GLSLD--FGP FAFMD-NFDP SYTPNHDD-D ML-RYSYKNQ PSVIWWNLVR LGESF--AQL IGIGDEKLID QAGDEFKTVF LNE-YKRLMS TRLGLKTQ-- KESD----FN ELF---SNLL

*gi_261192888_[Ajellomyces_d* IVRRNAKTVA AWQAYGFMNG VLNTDNTSIM GLSLD--YGP FAFLD-NFDP QYTPNHDD-H LL-RYSYKNQ PSVIWWNLVR LGESL--GEL MGAGDEKLID NTGEEYKTVF LNE-YKRLMS ARLALKAP-- KESD----FQ ELF---SELL

*gi_119196335_[Coccidioides_* IVRRNAKTVA AWQAYGFMNG VLNTDNTSIF GLSLD--YGP FAFMD-NFDP NYTPNHDD-E LL-RYSYRNQ PSIIWWNLVR LGESF--GEL IGAGDETIID NASKEYRTVF LNE-YKRLMT ARLGLKTQ-- KDSD----FE KLF---SDLL

*gi_164428165_[Neurospora_cr* IIRRNALTVA KWQIYGFMNG VLNTDNTSIM GLSID--FGP FAFMD-NFDP NYTPNHDD-F AL-RYSYRNQ ATIIWWNLVR LGEAL--GEL IGAGPHKLIT QAGEEFKAVF MGE-FKRLFT ARLGLKTY-- KESD----FD SLF---DSLL

*gi_145608380_[Magnaporthe_o* ICRRNAITVA HWQAYGFMNG VLNTDNTSII GLSMD--YGP FAFVD-VFDP SYTPNHDD-H AL-RYSYRNQ PTIIWWNLVR LGEAL--GEL LGAGAEALIT QAGTEYRAVF LAE-YKRLMC ARLGLRTH-- KDSD----FD KLF---SELL

*gi_19115652_[Schizosaccharo* VAYRNAKTVA KWQAYGFMNG VLNTDNTSIL GLSID--YGP FGFLD-VYNP SFTPNHDD-V FL-RYSYRNQ PDIIIWNLSK LASAL--VEL IGACDSEVFE KIVEEYKNIV QND-FYDLMF KRVGLP---- SDSS----NK ILI---TDLL

*FMP40_YEAST_gi_6325034_[Sac* VVSLNANTVA HWQAYGFANG VLNTDNTSIM GLTID--YGP FAFLD-KFEP SFTPNHDD-T AK-RYSFANQ PSIIWWNLQQ FAKDL--ACL LGPEA----- ---------- ---------- ---------- ---------- ----------

*gi_50303343_[Kluyveromyces_* IVTLNAECVA YWQAYGFCNG VLNTDNTSIL GLSID--FGP FGFMD-KFQP QYTPNHDD-Y NL-RYSFANQ PTVIWWNLIK LAEAL----- ---------- ---------- ---------- ---------- ---------- ----------

*gi_302308501_[Ashbya_gossyp* VVNLNAECVA YWQAYGFCNG VLNTDNTSLM GLSMD--FGP FSFMD-KYRP EYTPNHDD-I EG-RYSFSNQ PYVIWWNLTK FAEAL--VLL LGSGENALVN CAANEYRFRY HVK-YAEIMA KRLGI----- ---------- ----------

*gi_290979991_[Naegleria_gru* IVRRTAKLCA DWMSVGFVHG VLNTDNMSIL GLTID--YGP FGFVD-YFSE DFVPNNSD-S DG-RYRYKNQ PAIVFWNLQK LMRAF--TPT LLPEE--YFA KVLNVYAPHF EHY-YLMNFR KKLGLIDG-- DSENLRNEDW ELI---EGFL

*gi_229593872_[Tetrahymena_t* ITKRTAQLVA KWQSVGFCHG VLNTDNMSIV GVTID--YGP FGFME-HFDK KHICNHSD-K EG-YYCYQNQ PSACKWNLLR LIEGI--KWA VNEEQ--AKE YVIQNFDKIY YDH-YYTLMR RKIGLFED-- LYEKNLQLDK KII---NNLM

*gi|145516136_[Paramecium_te* IVNRTAKLVA YWQCYGFCHG VLNTDNMSII GLTID--YGP FGFMD-YFNK NHICNNSD-K EG-RYSYANQ PQVCLWNLNR LSEAL--EDV IPKDQ--SKQ IIQDSYWVEY KKW-YYHIML RKFGLIDQ-- NQMEFTKSQC ILV---DSFF

*gi_294872672_[Perkinsus_mar* VVERTAKLVA KWQCVGFCHG VLNTDNMSIV GDTID--YGP YGFVE-AFQR DYICNTSD-T GG-RYTYEAQ PRICLWNCTK LAEAL--API LDPEK--STD ILRSTYGRVF MKE-YKRLMA MKLGLV-E-- EREG----DS DLV---ERLL

*gi_115916063_[Strongylocent* VVKSTAKMVA EWQCVGFCHG VLNTDNMSIL GITID--YGP YGFLD-GYDP DHICNTTD-D RG-RYTFTRQ AEMCRWNLGK FAEAL--SMC LPEEQ---SK AELELFDETF EEH-YITKMR KKLGLLQN-- NLPE----DR ALI---DSLH

*gi_167537910_[Monosiga_brev* VTRRTAALVA QWQCVGWCHG VLNTDNMSVL GLTID--YGP FGFME-QYDP NFICNRSD-D GG-RYDYQSQ PEICRWNLHR LADVL--VPH LPLER--ARD IIDRHYTRTF EQA-YMDGMR AKLGLL-Y-- PQGE----DQ ELI---KALF

*gi_256073786_[Schistosoma_m* VVKRTANLVA LWQTVGFCHG VLNTDNMSII GLTID--YGP FGFMD-QFTW DHISNTSD-P DG-RYSYAQQ PNICAWNCAR LAECL--IQA LIDQQRKFTN VLDTTYMSYF KSV-YLERMR KKLGLF---- KDEI----DA DLI---ENLF

*gi_159483357_[Chlamydomonas* VTRRTASLVA AWQCVGWCHG VLNTDNMSIV GVTID--YGP FGFLD-RYDP DFICNGSD-D SG-RYDYKSQ PDICRWNCER LAEAV--RAV LPEGR--GKR AVAEVFDAVY RKCVWRGALV CTLGAG-R-- AAVE----DE GLA---AALL

*gi_302845399_[Volvox_carter* VTRRTASLVA AWQCVGWCHG VLNTDNMSVV GVTLD--YGP FGFLD-RYDP DHICNGSD-D SG-RYDYKSQ PDICRWNCEK LAEAI--RTV LPEAR--GKR AVAETFDPVY RRT-YLGLMR RKLGLADIDA DAGE----DE MLV---SELL

*gi_298286503_[Ciona_intesti* VCVRTAALVA KWQCVGFCHG VLNTDNMSLL GLTID--YGP FGFMD-RFDP NFQCNNSD-N KG-RYVYKAQ PEICQWNLKK FAEAI--QEC LPLND--SLK VLEESYFPEY KQQ-YLSEMR KKLGLV-K-- NLPE----DE ALV---DSFL

*gi_221116553_[Hydra_magnipa* IVRRTAFMVA KWQCVGFCHG VLNTDNMSII GVTID--YGP FGFMD-YFNS DFICNASD-T NG-RYSYKKQ PEICKWNLLK LAEAI--KNA VPLDK--TKE IINEIYDSEF RES-YYKGMR EKLGLK-T-- NNVN----DE KLI---QNLL

*gi_340370931_[Amphimedon_qu* ICRLTGRLVA MWQCVGFCHG VLNTDNMSIV GVTID--YGP FGFLD-RYDP AHICNKSD-D GG-RYAFSKQ PSVCKWNLRK LSEAL--SPC LSTEK---AD EGLELYEMEF QQT-YLSKIR EKLGLVNK-- AFPE----DS DLV---EQFL

*gi_156406460_[Nematostella_* LVVKTARLVA QWQCVGFCHG VLNTDNMSIV GLTID--YGP FGFMD-AFDP QHICNDSDAD RG-RYRYGAQ PEICKWNLMK LGEAI--HDA LPVDQ--SLA ALEELYDKEY QGA-FLSKMR LKLGLLNK-- QQPE----DV DLI---EALF

*gi_195999240_[Trichoplax_ad* VVKATAKLVA LWQCVGFCHG VLNTDNMSIA GITID--YGP FGFLD-VYDP DYVCNASD-D GG-RYAFINQ PEACKWNLSK LAEAL--ASV LPLAD---SN PVLEKYNELF HKF-YLEKMR LKLGLIRK-- QLPG----DE YIL---SKLL

*gi_260794380_[Branchiostoma* IVHRTARLVA EWQCVGFCHG VLNTDNMSIL GLTID--YGP FGFLD-RYDA DNICNGSD-D GA-RYSYRNQ PEMCKWNCEK FSEAI--SEA LPTVL---SK PVLEEFDPKF SEH-YLSKM- ---------- ---------- ----------

*gi_319738592_[Xenopus_(Silu* ITKRTARLVA EWQCVGFCHG VLNTDNMSIV GLTID--YGP FGFID-RYDP EYICNGSD-N MG-RYAYNKQ PEICKWNLGK LAEAL--IPE LPLSI--SQS ILDDEYDAEF QNH-YMEKMR KKLGLVRL-- KLDD----DS HLV---SDLL

*gi_327273185_[Anolis_caroli* VTRRTARMVA EWQCVGFCHG VLNTDNMSIV GLTID--YGP FGFMD-RYDP EHICNGSD-N TG-RYAYNKQ PEVCKWNLGK LAEAL--DPE LPLEI--SIP ILEEEYDTEF GKH-YLQIMR KKLGLIQL-- QLAD----DD KLV---SDFL

*SELO_CHICK_gi_169234793_[Ga* ITKRTARLVA EWQCVGFCHG VLNTDNMSIV GLTID--YGP FGFMD-RYDP EHICNGSD-N TG-RYAYNRQ PEICKWNLGK LAEAL--VPE LPLEI--SEL ILEEEYDAEF EKH-YLQKMR KKLGLIQL-- ELEE----DS KLV---SELL

*gi_334347697_[Monodelphis_d* ITRRTARLVA DWQCVGFCHG VLNTDNMSIV GLTID--YGP FGFMD-RYDP DHVCNSSD-T TG-RYAYSKQ PEVCKWNLRK LAEAL--VPE LPLEL---SE PVLEEYDAEF DKR-YLHKMR QKLGLVQL-- QLEE----DR ELA---AALL

*SELO_BOS_gi_319803072_[Bos_* VTRRTARLVA EWQCVGFCHG VLNTDNMSIV GLTID--YGP FGFLD-RYDP DHVCNASD-T AG-RYSYSKQ PEVCKWNLQK LAEAL--DPA LPLEL--AEA ILAEEFDAEF GRH-YLQKMR RKLGLVQT-- EQEG----DG ALV---AQLL

*gi|81295807_[Mus_musculus]*  VTQRTARMVA EWQCVGFCHG VLNTDNMSIV GLTID--YGP FGFLD-RYDP DHICNASD-N AG-RYTYSKQ PQVCKWNLQK LAEAL--EPE LPLAL--AEA ILKEEFDTEF QRH-YLQKMR KKLGLIRV-- EKEE----DG TLV---AKLL

*SELO_HUMAN_gi_32880229_[Hom* VTRRTARMVA EWQCVGFCHG VLNTDNMSIL GLTID--YGP FGFLD-RYDP DHVCNASD-N TG-RYAYSKQ PEVCRWNLRK LAEAL--QPE LPLEL--GEA ILAEEFDAEF QRH-YLQKMR RKLGLVQV-- ELEE----DG ALV---SKLL

*gi_47600579_[Escherichia_co* LARREAWQMA -FCRTRFLRL TTSPSNIAMD GRLMD-FNGL SC-LF-PGDS PADFGYK-LR L-AELAKEPM VLMQGLSDLC LYIG--KYMF DPDA--RLKV EEIFQKTFHE AC-YYCYLEL LGIPGEF--I T------QKE IP---DILKE

*gi_338721443_[Equus_caballu* VTRRTARMVA EWQCVGFCHG VLNTDNMSIV GLTID--YGP FGFLD-RYDP DHVCNASD-N AG-RYTYSKQ PEVCKWNLQK LAEAL--EPE LPREL--GEA ILAEEFDAEF HRH-YLQKMR RKLGLVQA-- EQEE----DA VLV---AKLL

*gi_71003666_[Ustilago_maydi* VATRNAKTIG LWQVYGFMHG VMNTDNIALT GHTID--YGP YAFMD-LYDE SQICNHSD-G EG-RYAYRLQ PTMGVFAIRE LLNAV--APV VGFELDEYSA ELENVFTNTL LEQ-WKQAYR ARLGLQTE-- QADD----KS ALI---DPL-

*gi_58271330_[Cryptococcus_n* VIKKNAEMVA KWQVYGYMNG VINTDNVTLM GITID--YGP YAFMD-VFDQ KHISNQSD-P TG-MYAYRNQ VSRVQFALSK FIDSV--SPL L--------- ---------- ---------- ---------- ---------- ----------

310 320

....|....| ....|....| ....

*gi_308808097_[Ostreococcus_* RVLRVGGFDF TRAHRALGEI A---

*gi|298715152_[Ectocarpus_si* DLMAACRTDF TETWRALLDV PA--

*gi|323452602_[Aureococcus_a* ---------- ---------- ----

*gi_224011106_[Thalassiosira* PLLRGGRADW TLFWRQLTYV A---

*gi_219126041_[Phaeodactylum* YMLRHSRTDW TIFFRQLSYI T---

*gi_219124584_[Phaeodactylum* PLLRLSQIDW TLFFRQLT-- ----

*gi_145355852_[Ostreococcus_* GLMYESQADW TVTWRRLAEC A---

*gi_308813832_[Ostreococcus_* RLMYEDRADW TMTWRQLAEC ----

*gi_255074165_[Micromonas_sp* PLMRDSNVDY TLVFRQLAEC ----

*gi|323455901_[Aureococcus_a* ---------- ---------- ----

*gi_303290328_[Micromonas_pu* PLMAESEVDY IVLFRNLSGV ----

*gi|321463811_[Daphnia_pulex* DMMQQRRADF TQTFRQLGAI HLD-

*gi_196009079_[Trichoplax_ad* KMMKNQKADF TMTFRELSEI D---

*gi_156359336_[Nematostella_* QIMESSQADF TMTFRQLGNL T---

*gi_115638715_[Strongylocent* QMMEDTHSDF TTTFRQLGVI S---

*gi_291227954_[Saccoglossus_* KMMEDTRTDF TMTFRQLGNL T---

*gi_260794897[Branchiostoma_* KMMADTKADF TMTFRQLSEL S---

*gi_319738607_[Xenopus_(Silu* DLMASTRADF IMSFRQLSEI S---

*gi_194227089_[Equus_caballu* HLMEKTAADF TMTFRQLSEI T---

*SELO2_BOS_gi_297481447_[Bos* HLMEKTEADF TMTFRQLSEI T---

*gi_327274681_[Anolis_caroli* KLMEDSRADF TMTFRQLSEI S---

*SELO2_CHICK_gi_118085664_[G* KLMEDTKADF TMTFRQLSEI T---

*YDIU_ECOLI_gi_3183285_[Esch* SLMARERSDY TRTF------ ----

*gi_159480380_[Chlamydomonas* KMMYTDDADF TNTFRALS-- ----

*gi_302841364_[Volvox_carter* RLMYDDDADF TNTFRALCSI S---

*gi_115467830_[Oryza_sativa_* NNLAVDKVDY TNFFRLLSNV K---

*gi_293335415_[Zea_mays]*  SNMAVDKVDY TNFFRLLSNV N---

*gi_30684227_[Arabidopsis_th* NNMSVDKVDY TNFFRLLANV K---

*gi_224053020_[Populus_trich* NNMAVDKVDY TNFFRLLSNV K---

*gi_255544744_[Ricinus_commu* NNMAVDKVDY TNFFRLLSNI K---

*gi_225435594_[Vitis_vinifer* NNMAVDKVDY TNFFRLLSNI K---

*gi_168047679_[Physcomitrell* NNMAFDKVDY TNFFRSFSNL K---

*gi_302804871_[Selaginella_m* SNLAFDKVDY TNFFRALASV ----

*gi_254564783_[Pichia_pastor* DMLQKTKLDY NNFFVQLQE- ----

*gi_68484234_[Candida_albica* DVLYKIQTDF NLFFLKLQDL ----

*gi_154318896_[Botryotinia_f* DTMESLELDF NQFFRKLSSI P---

*gi_255931617_[Penicillium_c* DTLETLELDF NHFFRRLSGL T---

*gi_317029685_[Aspergillus_n* DTLEALELDF NHFFRRLSSV S---

*gi_327297586_[Trichophyton_* DTLEKLELDF NHFFRRLSGL T---

*gi_302510829_[Arthroderma_b* DTLEKLELDF NHFFRRLSGL T---

*gi_261192888_[Ajellomyces_d* DTLEALELDF NHFFRHLSSI S---

*gi_119196335_[Coccidioides_* DTLEALELDF NHFFRKLSLP R---

*gi_164428165_[Neurospora_cr* NTMEALELDY NLFFRRLSTL K---

*gi_145608380_[Magnaporthe_o* DTMEAAELDF NFFFRRLSSL ----

*gi_19115652_[Schizosaccharo* QILEDYELDM PNCFSFLS-- ----

*FMP40_YEAST_gi_6325034_[Sac* ---------- ----RDL--- ----

*gi_50303343_[Kluyveromyces_* ---------- ---------- ----

*gi_302308501_[Ashbya_gossyp* ---------- ---------- ----

*gi_290979991_[Naegleria_gru* AWMNENRADF TNFFRLLSNV K---

*gi_229593872_[Tetrahymena_t* DYMDSSGSEF TNFFRKLSQI ----

*gi|145516136_[Paramecium_te* DILHQSCTNF TKVFQILQQI EI--

*gi_294872672_[Perkinsus_mar* DTMENTAADF TNTFRALSTV K---

*gi_115916063_[Strongylocent* KAMQATRADF TNVFRSLSTL ----

*gi_167537910_[Monosiga_brev* TVMAKTSADF TNTFRLLSRF S---

*gi_256073786_[Schistosoma_m* NTMEKTGADF TNTFLALED- ----

*gi_159483357_[Chlamydomonas* SVMEATGADF TNTFRCLS-- ----

*gi_302845399_[Volvox_carter* TVMEETGADF TNTFRQ---- ----

*gi_298286503_[Ciona_intesti* NTMEETYADF TNSFRSLSVV S---

*gi_221116553_[Hydra_magnipa* YTMQQSASDF TNTFLILSGV S---

*gi_340370931_[Amphimedon_qu* DTLHETGCDF TNGFRKLNKV V---

*gi_156406460_[Nematostella_* ETMHKTGADF TNTFRALSRL G---

*gi_195999240_[Trichoplax_ad* FVFFWVGADF TNSFRCLNKL ----

*gi_260794380_[Branchiostoma* ---------- ---------- ----

*gi_319738592_[Xenopus_(Silu* ETMNITGSDF TNTFRVLSKF S---

*gi_327273185_[Anolis_caroli* ETMQVTGADF TNTFHFLSSF P---

*SELO_CHICK_gi_169234793_[Ga* ETMHLTGGDF TNIFYLLSSF S---

*gi_334347697_[Monodelphis_d* ETMRLTGADF TNTFCLLSSF S---

*SELO_BOS_gi_319803072_[Bos_* ETMHLTGADF TNSF------ ----

*gi|81295807_[Mus_musculus]*  ETMHLTGADF TNTFCVLSSF PADL

*SELO_HUMAN_gi_32880229_[Hom* ETMHLTGADF TNTFYLLSSF P---

*gi_47600579_[Escherichia_co* LVNSFVALLN KYYEKSH--- ---

*gi_338721443_[Equus_caballu* ETMHLTGADF TNTFYLLSSF P---

*gi_71003666_[Ustilago_maydi* -CLVLTDLDF STTLRRLCQL P---

*gi_58271330_[Cryptococcus_n* ---------- ---------- ----
