# Supplementary material for: A Novel Protein Kinase-Like Domain in a Selenoprotein, Widespread in the Tree of Life
Source: PLoS One. 2012 Feb 16;7(2):e32138. doi: 10.1371/journal.pone.0032138 (PMC3281104; doi:10.1371/journal.pone.0032138)
Supplement: Figure S4 — Multiple sequence alignment (MUSCLE) of mchC proteins, with human SELO and Escherichia coli ydiU added. Identifiers: NCBI gi numbers. (RTF) [file pone.0032138.s004.rtf]

                               10        20        30        40        50        60        70        80        90       100       110       120       130       140       150       160       170                         
                      ....|....|....|....|....|....|....|....|....|....|....|....|....|....|....|....|....|....|....|....|....|....|....|....|....|....|....|....|....|....|....|....|....|....|
289662952_Xanthomona  FLVDAYGSTGIASYGG-----SGRAGVIN---GYQVKGIGVTPFVDPDADWTHSHGSLLLQEGVRELVFSRVAAELFPFGAIESVALIELQQNITDDT--------GRSQRTALLVR--PFELRPCHFQRALGFRPNQINLRHLD-DVLRVKSCVSIA------------ 
66768711_Xanthomonas  LGADRYGGSGGAIHGG-----SGRCGSDG---MLIAKGTGPTPLVSEAHDWSHSHGCLYLYDAIREAVASEILNAELPHGTVPIVAIIDAGFSLARTDA-------DEPERCAILIR--PAFLRLAHFERSLYFGTSGSANSDQFQDSLRVKDAIHFA----------DE 
30387011_EsCheriChia  FHAERYGGSGIQRNGG-----GARCGFDG---NYQVKGIGSNPLVGEGTDERHSNGALGAVHAIYEALWGEVLAQILPYSAVRVRAVLLTD--LYTEKAFERS---GRKSRRALLVR--EPVVRPAHFERAPYFQVKPEYSSLLIHDACRVRSVIHKLPGYLPVPPE-EI 
15808049_Klebsiella   FHAERYGGSGIQRNGG-----GARCGFDG---NYQVKGMGANPLVGVGTDGRHSNGALGAIHAIYEALWGEVLAQILPYSAVRVQAVLLTN--LYTDKAFDRS---HGKSRRALLVR--DPVVRPAHFERAPYFRAKPEYAGQLIHDARRVRSVIRMLPSNLPVPSE-GF 
253988821_Photorhabd  FYAERYGGDGISRNGG-----GARCGFDG---QWQVKGIGANPLIGK--DSKQVDGELTMTGAMLEVLWGSLMEKLLPFGAVPNVVVLLTDQAISGKKHAISF---RSQDLRTLLVR--EPTIRPAHFCRAPYYLPYAAMLSQ-QHDASRVENLISKLIGCLPHPLSMDE 
192362489_Cellvibrio  LQAERYGGLGVGINAG-----GARCGNLN---GVQIKGVGKNILAGESNDEWYSYGGLNLVDAIYEAIYSTLLNRIMPLGTVAIHGVILLGEKTAMLPGLDHLPIDQRRGYGALLVR--DICIRPAHFLKASSSRISIPN--DIISEEARVRRVNRNF------------ 
192359849_Cellvibrio  LFAERYGGNGVGTNGG-----GARTGNLE---NFQLKGVGKNILAANTTDDWHSYGGLNLVDAVIETISYLLLKNILPIGCVKIYGIIITGDETAYLPG-------GKIGPGAILVR--EKVTRPGHFLPCPAYNVKEIQ--DIPQDSYRLRYINKIL------------ 
192361021_Cellvibrio  LTAEQYGGDGVSKNGG-----GARCGNID---CFQLKGIGVNPLLGTYDRFGNSNGIYSVHEALREIIYTMLFDYIFPGKVVKILGLINLGESTHADPSF------DIRLPLVISVR--EKCVRPAHFFRAPHFEPRKEDRSRIVSDIARVRYAWMQL------------ 
192361262_Cellvibrio  MFAERYGGFGIGTNGG-----GARVVNIN---GMQIKGGGANALAGDGALRSHSYGGLDIQGAVKEIIYSRLLSKISPVGTQTIKGLILLDDTSALHN--------GNKAPSVLMVR--ETVVRPGHFLPCVNFRLKPEHRSLMRSDYSRVLGIYKSI------------ 
192359747_Cellvibrio  AQAEIYPG---GSNAG-----GSRSGNIA---GYHVKGMGANPLLGEVDYDWYSYGGLSFYEAALEAINTIILNAILPYGCVNCYAIIKTGDRTALHPTGKLL--DDNRGKGALLVR--EQCLRPAHFFPLENFVPVDRNQYLVEFDQERVSSLSQKL------------ 
260779283_Vibrio Cor  FMADQYGSRHEVCNGG-----SARCGING---NFQIKGIGRNPLISQNISESHSHGKLFIDEAISEAIWGEICHKHLPYGAIRTLAIIKTNTKHAFTYQDD-----TPNKHCALAVR--EMSVRPAHFERCTFFWPEKSYSFLRDNDANRVRKAVPYL------------ 
153825930_Vibrio Cho  FLADRYGNPGDAGNGG-----SARCGLNG---HFQVKGNGTNPLGAVNVDEGHSHGKLPLSEAVSEAIWSEICHKELPYGALRIIAIIRTSQTILTTNTFGD----VVEQPCALMIR--EVAIRPAHYEPALNFWPKPEFVRLRDANSKVLELAVNKL----------EN 
YDIU_ECOLI_3183285    YSGHQFGVWAGQLGDGRGILLGEQLLADGTTMDWHLKGAGLTPY------SRMGDGRAVLRSTIRESLASEAMHY-LGIPTTRALSIVTSDSPVYR----------ETAEPGAMLMRVAPSHLRFGHFE---HFYYRRESEKVRQLADFAIRHYWSHL------------ 
SELO_HUMAN_32880229   YCGHQFGQFAGQLGDGAAMYLGEVCTATGERWELQLKGAGPTPF------SRQADGRKVLRSSIREFLCSEAM-FHLGVPTTRAGACVTSESTVVRDVFYDGN---PKYEQCTVVLRVASTFIRFGSFE---IFKSADEHTGRAGPSVGRNDIRVQLL-DYVISSFYPEI
                            ==G-loop===                   K72                          E91 

                              180       190       200       210       220       230       240       250       260       270       280       290       300       310             
                      ....|....|....|....|....|....|....|....|....|....|....|....|....|....|....|....|....|....|....|....|....|....|....|....|....|....|....|....|.
289662952_Xanthomona  --------AR--NCPAVLSDFARRLGAQIATMYRLGWFHGGVYSSNFSVSAKLIDFGSSRFI-----IDREQRSYSQHGPKFGEEIQFASMLLRSWCYYWNRYA-MGHNIDYSVLIRELHCGYEEQLLTYPSPTLGEVVGM 
66768711_Xanthomonas  HAQLDAGNPN--GLGVDLSELYLRLAEQIAASRAHRLWTGRPTSDNITSNAQFLDFGGFRAV------PSWKRGTDGHRHFFGDEMRDVRHSLPSLAYFFKKYSRFDKAIANID---QLLIQIEHHMEAAFLHHCAEACAL 
30387011_EsCheriChia  DAEARTDPRI--YCIEGLCELARREAWQMAFCRT-RFLRLTTSPSNIAMDGRLMDFNGLSCL--FPGDSPADFGYKLRLAELAKEPMVLMQGLSDLCLYIGKYM-FDPDFTLAARL-KVEEIFQKTFHEACYYCYLELLGI 
15808049_Klebsiella   SGEARRNPRI--YCIEGLCELARREAWQMAFCRT-RFLRLTTSPSNIAMDGRLMDFNGLSCL--FPGDYPDNFGHQFRLAELVKEPMVLIQGLSDLCLYIGKYM-FDPDFTLVSRL-KVEETFQKTFHEACYYCYLEQLGI 
253988821_Photorhabd  EQWHNLSPEE--KAGHGLVELTSRLATQIAYCRT-RHLVMRTSPSNCDMSGRLLDFHGVRHA--FPADREQGIQSYIRYEKLNGDAQILLNGMLDLCFYLAKYIFGSAFQIYVQR--QIINAFNSSYQRTSWVENLRIAGF 
192362489_Cellvibrio  --YKTFTSVS--GYIKSMGQLLFNYANQFAFSRMARLSHGSISPSNISIDGRWLDLTNTTFL-----GGGHNIG---GKSSFYSEPDEVIDFFCEMVYTFSKYNKLKLNVN------ILTNYFFEQFDSFLRAHTCYVLGI 
192359849_Cellvibrio  --YKKFRDPR--ELAVALAKMASMIADQFGFSKIARIFHGSISASNLSIDGRWLDLTNCTFI-----NDVHNYV---GSTPFHAEGIHIVSILEEWIYTIRKYNNLRFNER------IILDYYASQLKSSCYKHIGYLFAI 
192361021_Cellvibrio  --SKELPHHQ--QAINLMYDFLSTSAEMFARAFIHRFAHGAISPSNLSITGKWLDLTNASFI-----NGCQNYQAAKDTIAFSRELDNVFLIANQFLVECEKYCQTSLEYHRG----QLYFYYFTRLLNEKLNNLPELFGL 
192361262_Cellvibrio  ---GKQSLLS--EFYTLIQHFLDKCADQLSFFRMARLSHNALTPSNICLDGRVLDTALCSFV-----VSGSNYG---QVTSYFEEASTPVLVAKEWFYLIHKFLTDTSVEE------HFLKLYEEKFYQYACINMGFLFGL 
192359747_Cellvibrio  --LNLLGGID--RLNDYIVNFYIKSAAQFSTAKIFRIFHGAMNYSNISFDGRWLDVATTAFV-----QGGSDYGVNRPLPSFYKEHISPLNYINVFYQEIDGIIPKE----------MIVKIYSAELKKHTIRSFIKIMGI 
260779283_Vibrio Cor  --SKLLGGMEDIPLGEVLNELINRLASQIAASRVKGIPHGSLTSSNISIDGRFLDFGTITAV-----PDFGNYVLANGVGAVWDDHELIESWLENLFDTVNHYSKGELTSNQIK---DLSYGFSKALCDYENRYLLDELGI 
153825930_Vibrio Cho  NEYQKTNSHK--PIFDVVERFVKRFATQVAVSRVKGFPHGSLTSSNIALDGRFLDLGTMSAI-----GDFSNVILTAGLGATWDDHLGIADWLWNFFYYLNKYSVHPLTQDEQE---QLVKHFLETLEEQENITTATECGI 
YDIU_ECOLI_3183285    -----ADDED--KYRLWFSDVVARTASLIAQWQTVGFAHGVMNTDNMSLLGLTLDYGPFGFLDDYEPGFICNHSDHQGRYSFDNQPAVALWNLQRLAQTLSPFVAVDALN-------EALDSYQQVLLTHYGERMRQKLGF 
SELO_HUMAN_32880229   QAAHASDSVQ--RNAAFFREVTRRTARMVAEWQCVGFCHGVLNTDNMSILGLTIDYGPFGFLDRYDPDHVCNASDNTGRYAYSKQPEVCRWNLRKLAEALQPELPLELGEA------ILAEEFDAEFQRHYLQKMRRKLGL 
                                                            H166   N171     D184                            E208                            
