# Supplementary material for: A Novel Protein Kinase-Like Domain in a Selenoprotein, Widespread in the Tree of Life
Source: PLoS One. 2012 Feb 16;7(2):e32138. doi: 10.1371/journal.pone.0032138 (PMC3281104; doi:10.1371/journal.pone.0032138)
Supplement: Table S1 — Structure predictions for SELO proteins. Assignments of human SELO protein (gi: 172045770), yeast (S. cerevisiae) FMP40 protein (gi: 3183490), E. coli ydiU protein (gi: 16129662) and E. coli mchC protein (gi: 47600579) to PDB, SCOP and Pfam using FFAS and HHpred methods. Top hits shown for each combination of method, query protein, and database. For Scop hits, d.144 denotes members of Protein kinase-like (PK-like) fold. (DOC) [file pone.0032138.s006.doc]

| **FMP40_YEAST, FFAS predictions** | | | | | | | | | | |
| --- | --- | --- | --- | --- | --- | --- | --- | --- | --- | --- |
| **Z Score** | | **HIT ID** | | | | | | **Hit name** | | **Organism** |
| **Pfam** | | | | | | | | | | |
| -6.490 | | PF06293.7 | | | | | | Lipopolysaccharide kinase (Kdo/WaaP) family  (PKinase clan) | | - |
| -5.850 | | PF00723.14 | | | | | | Glycosyl hydrolases family 15  (Six-hairpin glycosidase clan) | | - |
| -5.830 | | PF04655.7 | | | | | | Aminoglycoside/hydroxyurea antibiotic resistance kinase (PKinase clan) | | - |
| -5.410 | | PF00925.13 | | | | | | GTP cyclohydrolase II | | - |
| **SCOP** | | | | | | | | | | |
| -7.350 | | 1nd4 | | | d.144.1.6 | | | Aminoglycoside 3`-phosphotransferase IIa (Kanamycin kinase) | | *Klebsiella pneumoniae* |
| -5.700 | | 2bz1 | | | c.144.1.1 | | | GTP cyclohydrolase II, RibA | | *Escherichia coli* |
| -5.640 | | 1zyl | | | d.144.1.6 | | | RdoA kinase | | *Escherichia coli* |
| -5.610 | | 2ppq | | | d.144.1.6 | | | Homoserine kinase ThrB | | *Agrobacterium tumefaciens* |
| **PDB** | | | | | | | | | | |
| -7.680 | | | 1e8y | | | | | Phosphatidylinositol 3-kinase catalytic subunit | | *Homo sapiens* |
| -7.520 | | | 2rd0 | | | | | Phosphatidylinositol-4,5-bisphosphate 3-kinase | | *Homo sapiens* |
| -7.460 | | | 2wxf | | | | | Phosphatidylinositol-4,5-bisphosphate 3-kinase | | *Mus musculus* |
| -7.370 | | | 1e7u | | | | | Phosphatidylinositol 3-kinase catalytic subunit | | *Sus scrofa* |
| **FMP40_YEAST, HHpred predictions** | | | | | | | | | | |
| **Pfam** | | | | | | | | | | |
|  |  | | | **Pval** | | **HIT ID** | | | **HIT name** | **Organism** |
|  |  | | | 0.000009 | | pfam06293 | | | Lipopolysaccharide kinase (Kdo/WaaP) family (PKL clan) | - |
|  |  | | | 0.00018 | | pfam01636 | | | APH phosphotransferase (PKL clan) | - |
| **SCOP** | | | | | | | | | | |
|  |  | | | 0.00041 | | 1zyl | d.144.1.6 | | RdoA kinase | *Escherichia coli* |
|  |  | | | 0.0017 | | 2pul | d.144.1.6 | | Methylthioribose kinase MtnK | *Bacillus subtilis* |
|  |  | | | 0.00065 | | 2ppq | d.144.1.6 | | Homoserine kinase ThrB | *Agrobacterium tumefaciens* |

| **SELO_HUMAN, FFAS predictions** | | | | | | | | | | |
| --- | --- | --- | --- | --- | --- | --- | --- | --- | --- | --- |
| **Z Score** | **HIT ID** | | | | **Hit name** | | | | **Organism** | |
| **Pfam** | | | | | | | | | | |
| -7.220 | PF06293.7 | | | | | Lipopolysaccharide kinase (Kdo/WaaP) family (PKL clan) | | | | - |
| -5.920 | PF00657.15 | | | | | GDSL-like Lipase/Acylhydrolase (SGNH_hydrolase clan) | | | | - |
| -5.790 | PF07579.4 | | | | | Domain of Unknown Function (DUF1548) | | | | - |
| -5.330 | PF01163.15 | | | | | RIO1 family (PKinase clan) | | | | - |
| **SCOP** | | | | | | | | | | |
| -7.050 | 1zar | d.144.1.9 | | | | Rio2 serine protein kinase C-terminal domain | | | | *Archaeoglobus fulgidus* |
| -5.900 | 1nd4 | d.144.1.6 | | | | Aminoglycoside 3`-phosphotransferase IIa (Kanamycin kinase) | | | | *Klebsiella pneumoniae* |
| **PDB** | | | | | | | | | | |
| -6.130 | 1e7u | | | | | Phosphatidylinositol 3-kinase catalytic subunit | | | | *Sus scrofa* |
| **SELO_HUMAN, HHpred predictions** | | | | | | | | | | |
| **Pfam** | | | | | | | | | | |
|  |  | | | **Pval** | | **HIT ID** | | **Hit name** | | **Organism** |
|  |  | | | 0.000008 | | pfam06293 | | Kdo (PKinase clan) | | - |
|  |  | | | 0.00041 | | pfam01636 | | APH phosphotransferase (PKinase clan) | | - |
|  |  | | | 0.00017 | | pfam07579 | | DUF1548 | | - |
| **SCOP** | | | | | | | | | | |
|  |  | | 0.0024 | | | 1zyl | d.144.1.6 | RdoA kinase | | *Escherichia coli* |

| **ydiU *E. coli,* FFAS predictions** | | | | | | | | |
| --- | --- | --- | --- | --- | --- | --- | --- | --- |
| **Z Score** | **HIT ID** | | **Hit name** | | | | | **Organism** |
| **Pfam** | | | | | | | | |
| -9.070 | PF06293.7 | | | Lipopolysaccharide kinase (Kdo/WaaP) (PKinase clan) | | | - | |
| -7.530 | PF06176.4 | | | Lipopolysaccharide core biosynthesis protein (WaaY)  (PKinase clan) | | | - | |
| -6.940 | PF05165.5 | | | GGDN family  (Nucleotide cyclase clan) | | | - | |
| -6.250 | PF00657.15 | | | GDSL-like Ligase/Acylhydrolase (SGNH_hydrolase clan) | | | - | |
| -5.680 | PF01163.15 | | | RIO1 family (PKinase clan) | | | - | |
| **SCOP** | | | | | | | | |
| -8.680 | 1zar | d.144.1.9 | | Rio2 serine protein kinase C-terminal domain | | | *Archaeoglobus fulgidus* | |
| -6.970 | 1zyl | d.144.1.6 | | RdoA kinase | | | *Escherichia coli* | |
| -6.960 | 1xws | d.144.1.7 | | Proto-oncogene serine/threonine-protein kinase Pim-1 | | | *Homo sapiens* | |
| **ydiU *E. coli,* HHpred predictions** | | | | | | | | |
| **Pfam** | | | | | | | | |
|  |  | **Pval** | | **HIT ID** | | **Hit name** | **Organism** | |
|  |  | 0.0000025 | | pfam06293 | | Lipopolysaccharide kinase (Kdo/WaaP) (PKinase clan) | - | |
|  |  | 0.00084 | | pfam08757 | | CotH | - | |
|  |  | 0.00071 | | pfam01636 | | APH phosphotransferase (PKL clan) | - | |
| **SCOP** | | | | | | | | |
|  |  | 0.00011 | | 1zyl | d.144.1.6 | RdoA kinase | *Escherichia coli* | |
|  |  | 0.00038 | | 2ppq | d.144.1.6 | Homoserine kinase ThrB | *Agrobacterium tumefaciens* | |

| **mchC *E. coli,* FFAS predictions** | | | | | | | | | | | | | |
| --- | --- | --- | --- | --- | --- | --- | --- | --- | --- | --- | --- | --- | --- |
| **Pfam** | | | | | | | | | | | | |  |
| -8.240 | | PF02696 | | | | Lipopolysaccharide kinase (Kdo/WaaP) family (PKinase clan) | | | | | - | |  |
| -7.050 | | PF06176 | | | | Lipopolysaccharide core biosynthesis protein (WaaY) (PKinase clan) | | | | | - | |  |
| -6.820 | | PF01163 | | | | RIO1 family (PKinase clan) | | | | | - | |  |
| **SCOP** | | | | | | | | | | | | |  |
| -8.190 | | 1zar | d.144.2 | | Rio2 serine protein kinase C-terminal domain | | | | | *Archaeoglobus fulgidus* | | |  |
| -8.040 | | 2ppq | d.144.1.6 | | Homoserine kinase ThrB | | | | | *Agrobacterium tumefaciens* | | |  |
| -7.110 | | 2pul | d.144.1.6 | | Methylthioribose kinase MtnK | | | | | *Bacillus subtilis* | | |  |
| **PDB** | | | | | | | | | | | | |  |
| -7.830 | | 2ppq | | | Homoserine kinase | | | | | *Agrobacterium tumefaciens* | | |  |
| -7.100 | | 2olc | | | Methylthioribose kinase | | | | | *Bacillus subtilis* | | |  |
| **mchC *E. coli,* HHpred predictions** | | | | | | | | | | | | |  |
| **SCOP** | | | | | | | | | | | | |  |
|  |  | | | **Pval** | | | **HIT ID** | | **Hit name** | | | **Organism** |  |
|  |  | | | 0.0031 | | | 1phk | d.144.1.7 | Phosphorylase kinase | | | *Oryctolagus cuniculus* |  |
|  |  | | | 0.00061 | | | 2ppq | d.144.1.6 | Homoserine kinase ThrB | | | *Agrobacterium tumefaciens* |  |
|  |  | | | 0.0055 | | | 2pul | d.144.1.6 | Methylthioribose kinase MtnK | | | *Bacillus subtilis* |  |
| **PDB** | | | | | | | | | | | | |  |
|  |  | | | 0.0041 | | | 3lzh | | Aminoglycoside phosphotransferase | | | *Enterococcus casseliflavus* |  |
|  |  | | | 0.011 | | | 2pyw | | 5-methylthioribose kinase | | | *Arabidopsis thaliana* |  |
