# Supplementary material for: A Novel Protein Kinase-Like Domain in a Selenoprotein, Widespread in the Tree of Life
Source: PLoS One. 2012 Feb 16;7(2):e32138. doi: 10.1371/journal.pone.0032138 (PMC3281104; doi:10.1371/journal.pone.0032138)
Supplement: Table S2 — Bacterial strains possessing mchC genes. (DOC) [file pone.0032138.s007.doc]

| Strain | number of *mchC* genes | *mchD* identified | mchC sequence identity to mchC from *E. coli* CFT073 | pathogenicity |
| --- | --- | --- | --- | --- |
| *Cellvibrio japonicus* Ueda107 | 5 |  | 27-35 |  |
| *Escherichia coli* 042 | 1 | y | 99 | human, persistent diarrhea |
| *Escherichia coli* 83972 | 1 | y | 100 | human, urinary tract infections |
| *Escherichia coli* CFT073 | 1 | y | 100 |  |
| *Escherichia coli* MS 45-1 | 1 | y | 100 |  |
| *Escherichia coli* Nissle 1917 | 1 | y | 100 |  |
| *Klebsiella pneumoniae* RYC492 | 1 | y | 73 | human, pneumonia and urinary tract infections |
| *Photorhabdus asymbiotica* subsp. asymbiotica ATCC 43949 | 1 | y | 42 | entomopathogenic, in mutaualistic association with nematodes, yet causes wound infection in humans |
| *Vibrio cholerae* AM-19226 | 1 | y | 30 | human, diarrhea |
| *Vibrio cholerae* MZO-2 | 1 | y | 30 | human, diarrhea |
| *Vibrio coralliilyticus* ATCC BAA-450 | 1 | y | 37 |  |
| *Vibrio furnissii* NCTC 11218 | 1 | y | 35 |  |
| *Vibrio harveyi* ATCC BAA-1116 | 1 | y | 46*short align |  |
| *Xanthomonas campestris* pv. campestris str. 8004 | 1 |  | 34 | plants, Black rot and citrus canker |
| *Xanthomonas campestris* pv. vasculorum NCPPB702 | 1 |  | 31 |  |
| *Xanthomonas gardneri* ATCC 19865 | 1 |  | 30 |  |
| *Xanthomonas oryzae* pv. oryzicola BLS256 | 1 |  | 34 | rice, Bacterial streak disease |
| *Xanthomonas vesicatoria* ATCC 35937 | 1 |  | 36 |  |
